# An Adaptive Refined Composite Multiscale Differential Symbolic Entropy Rooted in LSC-SAO and Its Application in Fault Diagnosis

**DOI:** 10.3390/e28060624

**Published:** 2026-06-01

**Authors:** Min Mao, Jingzong Yang, Chao Zhou, Chengjiang Zhou, Xuefeng Li

**Affiliations:** 1 Faculty of Information Engineering, Quzhou College of Technology, Quzhou 324000, China; 145465@qzct.edu.cn; 2 School of Big Data, Baoshan University, Baoshan 678000, China; 3 School of Information Science and Technology, Yunnan Normal University, Kunming 650500, China; 210007@ynnu.edu.cn; 4 College of Automobile and Traffic Engineering, Nanjing Forestry University, Nanjing 210037, China; lixuefeng@njfu.edu.cn

**Keywords:** snow ablation optimizer, fault diagnosis, adaptive refined composite multiscale differential symbolic entropy, logistic sine cosine, adaptive support vector machine

## Abstract

Accurate fault diagnosis of rotating machinery is critical for ensuring the reliability of the energy, industrial, and transportation sectors. However, conventional methods face significant challenges, including the susceptibility of the Snow Ablation Optimizer (SAO) to local optima, the instability of Multiscale Differential Symbolic Entropy (MDSE) with short time series, and the non-adaptability of Support Vector Machine parameters. To address these issues, this study proposes a parameter-adaptive fault diagnosis framework integrating an improved SAO with Adaptive Refined Composite Multiscale Differential Symbolic Entropy (Adaptive-RCMDSE). First, the Logistic Sine Cosine strategy (LSC) is introduced to enhance SAO’s global search capability, forming the LSC-SAO algorithm. Subsequently, an Adaptive-RCMDSE method is developed wherein LSC-SAO optimizes the control parameter to significantly improve feature stability for short time series. Furthermore, an Adaptive Support Vector Machine (Adaptive-SVM) model is constructed, employing LSC-SAO to automatically tune the penalty factor and kernel parameters for precise fault identification. Finally, validation is performed on gearbox, ball bearing, and axle box bearing datasets. Results indicate that the proposed method achieves superior diagnostic performance, with average accuracies of 99.70%, 99.29%, and 99.28%, respectively, outperforming existing methods. This work provides an effective and robust solution for intelligent health monitoring of rotating machinery.

## 1. Introduction

The reliable operation of mechanical equipment critically depends on the structural integrity of key components, such as gearboxes and bearings, which are susceptible to wear, fractures, and lubrication failures during prolonged operation [1]. Undetected faults in these components can lead to unexpected downtime, economic losses, and safety hazards, necessitating the development of efficient diagnostic methods to enhance maintenance efficiency and operational safety.

Against the backdrop of increasing industrial automation and precision control, traditional fault diagnosis methods are often limited by their reliance on individual expert experience or basic mathematical models [2]. These methods struggle to meet the stringent demands of modern industry regarding diagnostic accuracy and efficiency. In view of this, intelligent optimization algorithms have attracted increasing attention in the field of fault diagnosis due to their significant adaptability, self-learning capabilities, and excellent global search capabilities [3]. Intelligent optimization algorithms demonstrate significant potential in enhancing diagnostic performance. By simulating evolutionary mechanisms or biological behaviors, these algorithms can effectively address complex, nonlinear, and high-dimensional fault diagnosis problems [4]. These algorithms exhibit significant advantages in feature selection, pattern recognition, and fault classification, thereby injecting new vitality into the development of fault diagnosis techniques [5]. In recent years, several new algorithms with superior performance have been proposed successively, and their potential application in fault diagnosis continues to be explored. For example, the Rime Optimization Algorithm (RIME) [6], Newton–Raphson-based Optimizer (NRBO) [7], Crested Porcupine Optimizer (CPO) [8], and Snow Ablation Optimizer (SAO) [9] have been proposed. Notably, the CPO algorithm proposed by Abdel-Basset et al. draws inspiration from the defensive behavior of the crested porcupine (a large rodent) and can be applied to solve various optimization problems, particularly large-scale ones. The SAO algorithm proposed by Deng et al. is inspired by the phenomena of snow sublimation and melting and can be applied to solve various optimization problems. This algorithm simulates Brownian motion to search for potential regions in the solution space. However, despite significant advances in intelligent optimization algorithms for fault diagnosis, further optimization and enhancement are required regarding convergence speed, diagnostic accuracy, generalizability, and real-time performance [10]. To address these limitations, researchers have improved and optimized traditional intelligent optimization algorithms, thereby enhancing their optimization capabilities and efficiency. Examples of such techniques include opposition-based learning [11], mutation strategies [12], random walk strategies [13], and chaotic mapping [14]. Most notably, chaotic mapping is a method that leverages the properties of chaos theory to generate sequences exhibiting randomness, ergodicity, and aperiodicity [15]. It is frequently employed in optimization algorithms to enhance search capabilities and avoid local optima. Notable examples include Logistic mapping, Sine mapping, Tent mapping, and Cosine mapping. To address this, this paper proposes the use of a hybrid chaotic mapping method, Logistic Sine Cosine (LSC), to enhance intelligent optimization algorithms and applies it to feature extraction and fault classification to comprehensively improve fault diagnosis performance.

Feature extraction is a critical step in fault diagnosis and condition monitoring, designed to extract relevant information from raw signals that effectively characterize the health status of equipment. Common fault features mainly include time-domain, frequency-domain (e.g., those based on spectral kurtosis), and time–frequency-domain features [16]. Time-domain features are widely used owing to their advantages of intuitiveness, simplicity of calculation, real-time capability, versatility, lack of need for complex preprocessing, ease of hardware implementation, and direct correlation with faults. These features include those based on statistics, waveform analysis, fractal dimension, and entropy [17]. Among these, statistical features mainly include the mean, variance, and standard deviation. Waveform-based features include the waveform factor, peak factor, and pulse factor, among others [18]. Features based on fractal dimension include the Higuchi Fractal Dimension and the Katz Fractal Dimension [19]. Inspired by the physics-guided modeling paradigm, Chen et al. embedded physical principles into the feature learning process to guide the model in extracting more interpretable and robust representations that are consistent with underlying mechanical failure mechanisms [20]. The connection between entropy-based feature extraction and physics-guided modeling lies in using entropy as a physically interpretable metric to quantify the system’s increasing disorder, which directly reflects the underlying degradation process governed by physical laws. Commonly used metrics include Shannon Entropy, Sample Entropy, Approximate Entropy, Dispersion Entropy, Fuzzy Entropy, and Permutation Entropy [21]. Furthermore, some new entropies have been proposed successively. Wang et al. proposed Diversity Entropy (DivE), which utilizes the cosine similarity distribution between adjacent orbits to track internal pattern changes, yielding better performance in complexity estimation. Subsequently, it was extended to multiscale analysis, known as Multiscale Diversity Entropy (MDivE), to achieve comprehensive feature description by combining coarse-graining [22]. Rostaghi et al. proposed Fuzzy Dispersion Entropy (FDE), which combines the advantages of fuzzy theory and dispersion entropy to capture the uncertainty and dynamic changes present in time-series data more effectively [23]. Similarly, Diggans et al. proposed the Boltzmann–Shannon Interaction Entropy (BSIE). The BSIE introduces several improvements over traditional entropy estimators, including effectiveness on sparse samples and appropriate consideration of the impact of extreme outliers [24]. Particularly noteworthy is the Differential Symbolic Entropy (DSE) proposed by Yao et al. In the nonlinear complexity detection of chaotic logistic series, DSE effectively captures complexity variations in response to changes in the chaotic features of the logistic map [25]. To effectively extract nonlinear symbolic dynamics from complex systems exhibiting different structural or dynamical information, the control parameter within the differential symbolic transformation must be adjusted accordingly. To this end, this paper proposes utilizing an improved intelligent optimization algorithm to achieve the adaptive setting of its control parameter. Similarly, an adaptive DSE is extended to multiscale analysis, resulting in methods such as Adaptive Multiscale Differential Symbolic Entropy (Adaptive-MDSE), Adaptive Hierarchical Differential Symbolic Entropy (Adaptive-HDSE), Adaptive Composite Multiscale Differential Symbolic Entropy (Adaptive-CMDSE), Adaptive Time-Shift Multiscale Differential Symbolic Entropy (Adaptive-TSMDSE), and Refined Composite Multiscale Differential Symbolic Entropy (Adaptive-RCMDSE). These methods can be directly applied to fault feature extraction without the need for complex preprocessing.

Machine learning methods, with their excellent data processing and pattern recognition capabilities, are widely used and recognized in the field of fault diagnosis [26]. These methods include, but are not limited to, Extreme Learning Machine (ELM), Random Forest (RF), Decision Tree (DT), Radial Basis Function (RBF), and Support Vector Machine (SVM) [27]. For bearing fault diagnosis with small datasets, simulation-driven machine learning significantly reduces dependency on measured fault data by training models with high-fidelity synthetic vibration signals [28]. The zero-fault-shot learning framework further integrates physical models with hybrid algorithms, enabling cross-device fault classification without target machine fault data by projecting signals into an invariant feature space [29]. Although these approaches offer promising new paradigms for fault classification, a systematic analysis of their adaptability to small datasets remains lacking in existing research. Furthermore, deep learning methods are also widely used in fault diagnosis, including Long Short-Term Memory (LSTM), Convolutional Neural Network (CNN), Probabilistic Neural Network (PNN), Recurrent Neural Network (RNN), and their variants, Autoencoders, Deep Belief Network (DBN), and the Attention Mechanism (AM) [30]. However, different algorithms exhibit varying performance in fault diagnosis applications, with significant differences in their applicable scenarios, performance characteristics, advantages, and disadvantages. For instance, deep learning methods excel at handling nonlinear problems. However, they typically require large amounts of data, consume substantial computational resources, and involve complex training processes that are prone to overfitting. Moreover, these models exhibit lower transparency and interpretability [31]. The classification performance of ELM may be affected by randomly initialized parameters, leading to insufficient stability, and it may also suffer from overfitting and poor interpretability when dealing with complex data [32]. RF performs well with high-dimensional data but offers limited interpretability [33]. DT is prone to overfitting and poor generalizability, leading to reduced diagnostic accuracy on new data [34]. Although data-driven methods have been extensively applied in fault classification and Remaining Useful Life (RUL) prediction, their performance typically relies on abundant training data [35]. To address the issue of data scarcity, Yin et al. proposed a Fault Evolution Knowledge-driven Adversarial Meta-Learning (FEK-AML) method, thereby establishing a physics-guided learning framework [36]. SVM offers significant advantages in fault diagnosis, including its potent classification and generalization abilities, robustness, and proficiency in handling high-dimensional data, rendering it an effective tool for addressing complex fault diagnosis problems. However, despite its superior performance with small samples, SVM entails a relatively complex parameter selection process. To address this, Wang et al. introduced Bayesian optimization to search for the optimal penalty factor $C_{Best}$ and the optimal kernel parameter $\gamma_{Best}$ of SVM, thereby establishing an optimal Bayesian SVM model [37]. Furthermore, researchers have integrated the improved intelligent optimization algorithm with SVM in an organic manner. For instance, Liu et al. proposed a Good Point Set and Differential Evolution-Adaptive Honey Badger Algorithm (GD-AHBA) optimization method to enhance the performance of SVM and constructed the GD-AHBA-SVM model [38]. This paper proposes applying LSC-SAO to the parameter selection of SVM, thereby effectively constructing an adaptive SVM (Adaptive-SVM) model.

The implementation procedure outlined in this paper is as follows: First, LSC chaotic mapping is utilized to optimize the SAO algorithm, resulting in the LSC-SAO algorithm, which enhances its solution accuracy and global search capability for the objective function. Consequently, an adaptive fault feature extraction method utilizing Adaptive-RCMDSE and an adaptive fault classification recognition method employing Adaptive-SVM are proposed. During the feature extraction phase, the LSC-SAO algorithm is employed to adaptively determine the optimal control parameter $\alpha_{Best}$ of RCMDSE, effectively enhancing the stability and accuracy of feature extraction. In the classification stage, employing 5-fold cross-validation fault classification accuracy as the objective function, the LSC-SAO algorithm adaptively adjusts the optimal penalty factor $C_{Best}$ and the optimal kernel parameter $\gamma_{Best}$ of SVM, achieving precise classification of different types of faults. Adaptive-RCMDSE and Adaptive-SVM adaptively adjust their key parameters based on mutual feedback. This closed-loop optimization ensures that the extracted features are optimally suited for diagnostic tasks, significantly improving generalizability compared to standard combination methods.

The principal innovations and contributions of this paper are outlined below:

1. An intelligent optimization algorithm based on LSC-SAO is proposed. By incorporating chaotic mapping based on Logistic Sine Cosine, the global search capability of the SAO algorithm is enhanced, effectively preventing it from becoming trapped in local optima. This improves the accuracy of the objective function’s solution and the precision of parameter identification. This algorithm can be widely applied to adaptive fault feature extraction and classification recognition.
2. An adaptive fault feature extraction method based on Adaptive-RCMDSE is proposed. On the one hand, the LSC-SAO algorithm is employed to adaptively determine the optimal control parameter $\alpha_{Best}$ of Adaptive-RCMDSE, thereby accommodating various fault feature categories. On the other hand, by accurately extracting nonlinear symbolic dynamics from fault vibration signals, the stability and accuracy of DSE are significantly enhanced. This process refines the generation of coarse-grained sequences, strengthens the ability to handle short-time sequences, and effectively extracts fault features. Adaptive-RCMDSE is an efficient fault feature extraction method that demonstrates significant application value in fault identification and diagnosis.
3. An adaptive fault classification method based on Adaptive-SVM is proposed. By utilizing the LSC-SAO algorithm to adaptively determine the optimal penalty factor $C_{Best}$ and kernel parameter $\gamma_{Best}$ of Adaptive-SVM, precise classification of various fault types is achieved, fully demonstrating the accuracy and reliability of Adaptive-SVM in handling small-sample fault classification problems.

The paper is organized as follows: Section 2 introduces fault feature extraction and classification recognition methods. Specifically, Section 2.1 briefly introduces the theory of the SAO intelligent optimization algorithm and introduces the improvement of the SAO algorithm using chaotic mapping based on LSC. Section 2.2 briefly introduces the theory of Differential Symbolic Entropy, the construction process of the Refined Composite Multiscale Differential Symbolic Entropy, and the fault feature extraction method based on Adaptive-RCMDSE. Section 2.3 briefly introduces the process of improving the Support Vector Machine using the LSC-SAO algorithm and constructs an adaptive fault classification model based on Adaptive-SVM. Section 3 provides a brief overview of the novel fault diagnosis method proposed in this paper. In Section 4, the superiority of the proposed method is validated using the CEC2022 test functions, the planetary gearbox dataset from Beijing University of Technology (BJUT) and Beijing Jiaotong University (BJTU), the ball bearing dataset from Hanoi University of Science and Technology (HUST), and the High-speed Train Bogie Fault (HTBF) dataset from Southwest Jiaotong University (SWJTU). This section provides a detailed discussion of the experimental procedures for each stage and the comparative analysis results of the various methods. Section 5 concludes the paper by summarizing the research findings.

## 2. Fault Feature Extraction and Classification

### 2.1. Optimization of Key Parameters Based on LSC-SAO

(1) Population initialization. The SAO algorithm performs initialization by generating a population and searching for the optimal parameters for fault feature extraction and the parameter combination with the fault classification model (including the Differential Symbolic Entropy control parameter α and the combination of the SVM penalty factor C and kernel parameter γ), as shown in Equation (1):
  (1) $$\begin{array}{l} Z=L+\theta \times (U-L)\\ ={\left[\begin{array}{ccccc} Z_{1,1} & Z_{1,2} & \ldots & Z_{1,Dim-1} & Z_{1,Dim}\\ Z_{2,1} & Z_{2,2} & \ldots & Z_{2,Dim-1} & Z_{2,Dim}\\ \vdots & \vdots & \vdots & \vdots & \vdots \\ Z_{1,1} & Z_{2,2} & \ldots & Z_{N-1,Dim-1} & Z_{N-1,Dim}\\ Z_{N,1} & Z_{N,2} & \ldots & Z_{N,Dim-1} & Z_{N,Dim} \end{array}\right]}_{N\times Dim} \end{array}$$
  where Z represents the initial population; N represents the population size; Dim represents the dimensionality of the solution space; U represents the upper bound of the solution space; L represents the lower bound of the solution space; and θ represents the random number in the interval [0, 1].
(2) Exploration phase. Through the continuous exploration of the solution space, the SAO algorithm can accurately calculate the within-class and between-class scatter matrices of fault characteristics and the objective function of fault classification recognition rate, thereby identifying the control parameter α of differential symbolic entropy. The exploration phase takes place during the conversion of snow or liquid water into steam, and the position calculation formula is shown in Equation (2):
  (2) $$Z_{i}(t+1)=Elite(t)+BM_{i}(t)⊗(\theta_{1}\times (G(t)-Z_{i}(t))+(1-\theta_{1})\times (\bar{Z}(t)-Z_{i}(t)))$$
  where $Z_{i}(t)$ represents the i th individual in the i th iteration; Elite(t) represents the random individual selected from several elite sets; $BM_{i}(t)$ represents the random number contained in the Gaussian distribution based on Brownian motion; G(t) represents the current optimal solution; $\bar{Z}(t)$ represents the centroid position of the population; and $\theta_{1}$ represents a random number in the interval [0, 1]. The specific situation is described by Equation (3):
  (3) $$\left\{\begin{array}{c} \bar{Z}(t)=\frac{1}{N}\sum_{i=1}^{N}Z_{i}(t),\\ Elite(t)\in \left[G(t),Z_{\sec ond}(t),Z_{third}(t),Z_{c}(t)\right], \end{array}\right.$$
  where N represents the total number of individuals in the population; $Z_{\sec ond}(t)$ and $Z_{third}(t)$ represent the second best individual and the third best individual in the current population, respectively; individuals with fitness values ranking in the top 50% are considered leaders, and $Z_{c}(t)$ represents their centroid position. The specific situation is described by Equation (4):
  (4) $$Z_{c}(t)=\frac{1}{N_{1}}\sum_{i=1}^{N_{1}}Z_{i}(t),$$
  where $N_{1}$ represents the number of leaders, and the size is 1/2 of the population size.
(3) Exploitation phase. During the process of snow melting, the SAO algorithm continues to search the solution space around the current optimal solution to find a better solution, seeking enhanced solutions that continuously improve the accuracy of parameter identification for fault feature extraction and the fault classification model [9]. In the exploitation phase, the classic snowmelt model, the degree-day method [39], is used to reflect the process of snowmelt. The general form of the method is shown in Equation (5):
  (5) $$\left\{\begin{array}{c} M=DDF\times (T-T_{1}),\\ DDF=0.35+0.25\times \frac{e^{\frac{t}{t_{\max}}}-1}{e-1}, \end{array}\right.$$
  where M represents the snow melting rate; D represents the degree-day factor; T represents the daily average temperature; $T_{1}$ represents the base temperature, typically set to 0; and $t_{\max}$ represents the maximum number of iterations.

The specific formula for calculating the snowmelt rate is shown in Equation (6):

(6) $$M=(0.35+0.25\times \frac{e^{\frac{t}{t_{\max}}}-1}{e-1})\times e^{\frac{-t}{t_{\max}}}$$

The position update equation for the exploitation phase is shown in Equation (7):

(7) $$Z_{i}(t+1)=M\times G(t)+BM_{i}(t)⊗(\theta_{2}\times (G(t)-Z_{i}(t))+(1-\theta_{2})\times (\bar{Z}(t)-Z_{i}(t))),$$

where $\theta_{2}$ is a random number in the interval [−1, 1], and this parameter facilitates the interaction between individuals.

(4) LSC-SAO algorithm. Accurately solving the objective function is key to fault feature extraction and classification model parameter identification. Therefore, it is essential to improve the SAO algorithm’s accuracy and efficiency in solving the objective function [40]. Based on the SAO algorithm, this paper introduces the chaotic mapping method based on Logistic Sine Cosine and proposes the LSC-SAO algorithm. The algorithm aims to further improve the accuracy of the solution to the objective function, thereby enhancing the parameter identification accuracy. The algorithm uses Logistic and Sine maps as seed chaotic mappings, which are cascaded with the Cosine to form the Logistic Sine Cosine. The expression is shown in Equation (8):
  (8) $$\left\{\begin{array}{c} x_{i+1}=\cos\{\pi [F(\alpha ,x_{i})+G(1-\alpha ,x_{i})+\beta ]\}\\ F(\alpha ,x_{i})=4\alpha x_{i}(1-x_{i})\\ G(1-\alpha ,x_{i})=(1-\alpha )\sin(\pi x_{i}) \end{array}\right.$$
  where $F(\alpha ,x_{i})$ and $G(1-\alpha ,x_{i})$ are the known logistic and sine mappings, respectively; α and β are control parameters; and β (with a value of −0.5) is used to obtain the expression of the Logistic Sine Cosine mapping, as shown in Equation (9):
  (9) $$x_{i+1}=\cos\pi [4\alpha x_{i}(1-x_{i})+(1-\alpha )\sin(\pi x_{i})-0.5]$$
  where α∈[0,1], $x_{i}\in (0,1)$.

### 2.2. Fault Feature Extraction Based on Adaptive-RCMDSE

(1) Time series symbolization. A given time series N of length $X=\{x_{1},x_{2},\ldots ,x_{N}\}$ is processed through symbolic transformation to convert it into a symbolic sequence, as shown in Equation (10):
  (10) $$Z_{i}(x_{i})=\left\{\begin{array}{c} 1:(x_{i+1}-x_{i})>\alpha \sigma_{\Delta }\\ 2:(x_{i+1}-x_{i})>0\;and\;(x_{i+1}-x_{i})\leq \alpha \sigma_{\Delta }\\ 3:(x_{i+1}-x_{i})>-\alpha \sigma_{\Delta }\;and\;(x_{i+1}-x_{i})\leq 0\\ 4:(x_{i+1}-x_{i})\leq -\alpha \sigma_{\Delta } \end{array}\right.$$
  where $\sigma_{\Delta }$ represents the variance between two adjacent points; and α represents the control parameter.
(2) Phase space reconstruction. Each embedding $Z_{i}^{m}$ is calculated through Equation (11), as follows:
  (11) $$Z_{i}^{m}=\{z_{i},z_{i+\tau },\ldots ,z_{i+(m-1)\tau }\},i=1,2,\ldots ,N-(m-1)\beta$$
  where β represents the time delay; m represents the embedding dimension of phase space reconstruction; and N represents the length of time series.
(3) Calculate the differential pattern. If $z_{i}=v_{0},z_{i+d}=v_{1},\ldots ,z_{i+(m-1)d}=v_{m-1}$, then the time series $Z_{i}^{m}$ is mapped to the differential pattern $\pi_{v0,v1,\ldots ,v_{m-1}}(v=1,2,\ldots ,c)$. For $c^{m}$ possible differential mode $\pi_{v0,v1,\ldots ,v_{m-1}}(v=1,2,\ldots ,c)$, the corresponding probability is shown in Equation (12):
  (12) $$p(\pi_{v0,v1,\ldots ,v_{m-1}})=\frac{Number\{j|j\leq N-(m-1)\beta ,z_{j}^{m}\;counterpart\;\pi_{v0,v1,\ldots ,v_{m-1}}\}}{N-(m-1)\beta }$$
(4) Calculate the entropy value. According to the definition of Shannon entropy, the Differential Symbolic Entropy of time series X is calculated, as shown in Equation (13):
  (13) $$DSE(x,m,\alpha ,\beta )=-\sum p(\pi_{v0,v1,\ldots ,v_{m-1}})ln(p(\pi_{v0,v1,\ldots ,v_{m-1}}))$$

Relying solely on DSE for time series analysis may lead to an incomplete characterization of its complexity. Therefore, the effective information in a time series cannot be fully extracted. By contrast, the coarse-grained analysis of time series can achieve multi-scale signal analysis [41]. By combining coarse-graining analysis with DSE, a multi-scale entropy referred to as MDSE can be obtained. This allows for a more comprehensive analysis of vibration signals.

(5) Obtain the coarse-grained time series. For the original vibration signal {x(i),i=1,2,…,N}, after the transformation of Equation (14), the coarse-grained time series can be obtained:
  (14) $$y_{j}^{\tau }=\frac{1}{\tau }\sum_{i=(j-1)\tau +1}^{j\tau }x_{i},1\leq j\leq \left[\frac{N}{\tau }\right],\;i=1,2,\ldots ,N-\tau +1$$
  where τ represents the time scale; $y_{j}^{\tau }$ represents the new sequence obtained; j represents the index of the new sequence; and $\left[\cdot \right]$ represents rounded down.

**Figure 1:**
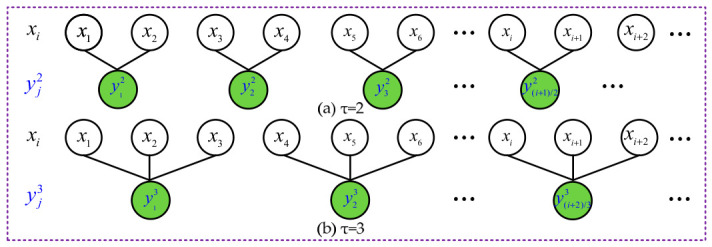
Schematic of the MDSE coarse-graining process.

When τ=1, the coarse-grained sequence is identical to the original signal. Figure 1 illustrates the coarse-graining process when τ=2 and τ=3.

(6) Calculate the MDSE. The differential symbolic entropy is calculated for each coarse-grained sequence $y_{j}^{\tau }$. Then, the differential symbolic entropies calculated from coarse-grained sequences at different scales τ are combined. This forms the Multiscale Differential Symbolic Entropy. The expression is shown in Equation (15):
  (15) $$MDSE(X,m,\alpha ,\beta ,\tau )=DSE\sum_{k=1}^{\tau }\left(y_{k}(j\right))$$

In the calculation of MDSE, the original signal is equally segmented and the average of each segment is calculated. This processing method has the advantages of reducing computational complexity and increasing execution speed. However, when segmenting data, the relationship between segmented data is not considered, resulting in insufficient information. In addition, variations in the initial point position may lead to certain deviations in the results.

(7) Calculate the RCMDSE. RCMDSE builds upon MDSE, further refining the preprocessing of the original signal. First, the original signal is continuously divided into small segments of length τ according to [1,τ]. The mean value of each segment is calculated sequentially. Then, these mean values are arranged sequentially into coarse-grained sequences, resulting in a total of τ coarse-grained sequences. To this end, the coarse-grained treatment in Equation (14) is improved, as shown in Equation (16). Finally, the RCMDSE is calculated, as shown in Equation (17).
  (16) $$y_{j,k}^{\tau }=\frac{1}{\tau }\sum_{i=(j-1)\tau +k}^{j\tau +k-1}x_{i},\begin{array}{cc} 1\leq j\leq \left[\frac{N}{\tau }\right], & 1\leq k\leq \tau \end{array}$$
  (17) $$RCMDSE=\frac{1}{\tau }\sum_{k=1}^{\tau }DSE(y_{j,k}^{\tau })$$

According to the findings of ref. [25], the control parameter a in the differential symbolic transformation should be adjusted accordingly. This adjustment is necessary to extract nonlinear symbolic dynamics from different structures or dynamics in complex systems.

(8) Calculate the Adaptive-RCMDSE. Using Equation (19) as the fitness function of the improved intelligent optimization algorithm, the optimal parameter $\alpha_{Best}$ is obtained. Subsequently, the Adaptive-RCMDSE is calculated accordingly. The calculation formula is shown in Equation (18):
  (18) $$Adaptive-RCMDSE_{\alpha_{Best}}=RCMDSE(X,m,\alpha_{Best},\beta ,\tau )=\frac{1}{\tau }\sum_{k=1}^{\tau }DSE(y_{j,k}^{\tau })$$

At the same time, the LSC-SAO algorithm is used to optimize it, with its objective function shown in Equation (19):

(19) $$fitness_{\alpha }=-\frac{trace(S\_B)}{trace(S\_W)}$$

where S_W represents the scatter matrix within the same fault category feature; S_B represents the scatter matrix between features of different fault categories; trace(S_W) is the trace of S_W; and trace(S_B) is the trace of S_B.

### 2.3. Fault Classification Method Based on Adaptive-SVM

The SVM is based on statistical learning theory and the principle of structural risk minimization. It has strong learning and generalization capabilities. The SVM is effective in handling problems such as small sample sizes, nonlinearity, and local minima, thereby enabling accurate classification [38].

(1) Optimization objective of SVM. The basic principle of SVM is to find an optimal separating hyperplane. The goal is to maximize the distance between different sample datasets and the hyperplane, and the core optimization problem is formulated in Equation (20):
  (20) $$\underset{w,b}{\min}\;\frac{1}{2}{\left\|w\right\|}^{2}+C\sum_{i=1}^{n}\xi_{i}\;s.t.\;y_{i}(w^{Τ}x_{i}+b)\geq 1-\xi_{i},\;\xi \geq 0\;\forall i=1,2,\ldots ,n$$
  where w and b are hyperplane parameters; C is the regularization parameter, which controls the penalty of classification error; and $\xi_{i}$ is the relaxation variable, allowing a small number of samples to violate the interval boundary.
(2) Selection of the kernel parameter. The Gaussian kernel, also known as a Radial Basis Function (RBF), is employed in this paper to map the original data into a high-dimensional space, thereby addressing the linearly inseparable classification problem. The core idea is to make the data linearly separable in high-dimensional space through nonlinear transformation, as shown in Equation (21):
  (21) $$K(Fea_{i},Fea_{j})=\exp(-\gamma {\left\|Fea_{i}-Fea_{j}\right\|}^{2})$$
  where $F{\mathrm{ea}}_{i}$ and $F{\mathrm{ea}}_{j}$ are the feature vectors of two samples; ${\left\|F{\mathrm{ea}}_{i}-F{\mathrm{ea}}_{j}\right\|}^{2}$ is the squared Euclidean distance between the two samples; and γ controls the width of the kernel parameter and determines the complexity of the decision boundary.
(3) Decision function. After training, the fault category of a new sample Fea is predicted, as shown in Equation (22):
  (22) $$f(Fea)=sign(\sum_{i=1}^{n}\alpha_{i}y_{i}\;K(Fea_{i},Fea)+b)$$
  where $\alpha_{i}$ denotes the Lagrange multiplier, which is nonzero only for the support vectors corresponding to $\alpha_{i}>0$.
(4) Fault classification model based on Adaptive-SVM. As can be seen from steps (1) and (2), the classification performance of SVM is closely related to the effective selection of parameters C and γ. To address this, the LSC-SAO algorithm is introduced in this study. By analyzing different types of faults, it adaptively selects key parameter combinations for the SVM, thereby effectively improving training speed, generalization ability, and applicability. In addition, 5-fold cross-validation is used in the Adaptive-SVM classification model. Its core goal is to evaluate the generalization performance of the model by dividing the dataset many times [42]. Assuming a specific fold in the cross-validation, the fault classification accuracy (i.e., performance metric) is given by Equation (23):
  (23) $$Accuracy_{Adaptive-SVM}=\frac{TP+TN}{TP+TN+FP+FN}$$
  where TP denotes True Positive; TN denotes True Negative; FP denotes False Positive; and FN denotes False Negative.
(5) Final performance of cross-validation. Assuming the validation result of the i th fold is $Metric_{i}$, the final performance is calculated as shown in Equation (24):
  (24) $$Final\_Metric=\frac{1}{5}\sum_{i=1}^{5}Metric_{i}$$

## 3. Fault Diagnosis Method Based on Adaptive-RCMDSE and Adaptive-SVM

To enable effective and accurate fault diagnosis, this paper proposes a novel fault feature extraction method, Adaptive-RCMDSE, and integrates it with an Adaptive-SVM for fault identification.

The specific implementation procedure is as follows:

1. Vibration signal sampling. To effectively address the challenges of limited data volume and data fragmentation, and to facilitate fault feature extraction, improve model robustness, and reduce training time, the original vibration signals are sequentially sampled. In this study, the overlap between adjacent samples is set to 3/4. Subsequently, the sampled signals undergo preprocessing.
2. Parameter setting for Adaptive-RCMDSE. The parameters are configured as follows: embedding dimension m=5, time delay β=1, and scaling factor τ=20. Additionally, the control parameter α is set within the range of [0, 1].
3. Fault feature extraction. Based on the objective function in Equation (19), control parameter $\alpha_{Best}$ is optimized using the LSC-SAO algorithm. The Adaptive-RCMDSE algorithm is employed to extract features from vibration signals under different fault conditions, generating fault feature vectors. All samples are calculated based on $\alpha_{Best}$ to form a feature matrix. The resulting feature matrix for various fault states has dimensions of 100 × 20, which is divided into a training set (80%) and a testing set (20%).
4. Parameter initialization. The SAO parameters are initialized as follows: a population size of 10 and a maximum of 10 iterations. The search ranges for the SVM penalty factor C and kernel parameter γ are set to (0,100] and (0,100], respectively.
5. Fault classification training based on Adaptive-SVM. The objective function of the LSC-SAO algorithm is defined as the fault classification accuracy determined through 5-fold cross-validation. The optimal parameters $C_{Best}$ and $\gamma_{Best}$ of the SVM are obtained through classification training.
6. Fault classification and identification. The optimal parameters, $C_{Best}$ and $\gamma_{Best}$, are input into the SVM to construct the optimal fault diagnosis model. The fault test sample set is input to enable accurate identification of different fault types.
7. Comparative experimental analysis. Through a detailed comparative analysis, the superiority of the proposed method is demonstrated.

**Figure 2:**
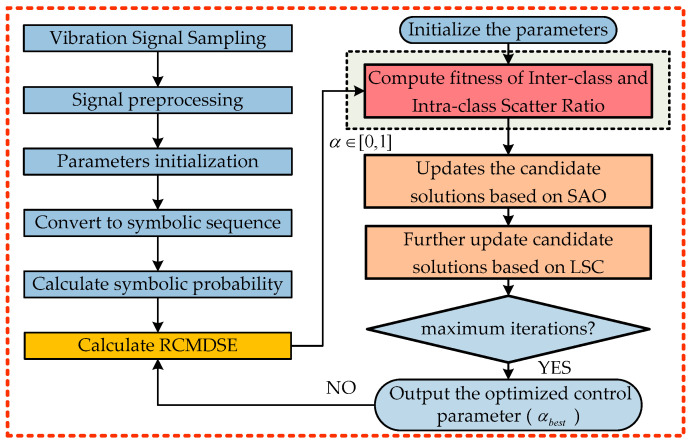
Flowchart of the feature extraction method based on Adaptive-RCMDSE.

**Figure 3:**
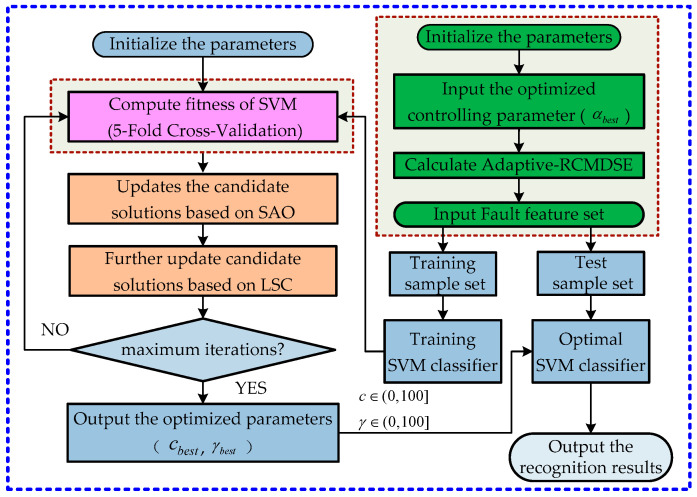
Flowchart of the adaptive fault diagnosis method based on Adaptive-SVM.

In summary, the proposed adaptive feature extraction method based on Adaptive-RCMDSE and the adaptive fault diagnosis method based on Adaptive-SVM are illustrated in Figure 2 and Figure 3, respectively.

## 4. Experimental Results and Analysis

To comprehensively validate the efficacy of the proposed method in fault feature extraction and classification, this study employs the CEC2022 test function alongside planetary gearbox [43], ball bearing [44], and axle box bearing [45,46] datasets for experimental analysis. Furthermore, the proposed method is compared in detail with other approaches. All experiments are conducted on a workstation with an Intel(R) Core (TM) i9-10900K CPU @3.70GHz and 32GB RAM, running MATLAB R2023a (64-bit). To ensure the accuracy and fairness of all experimental results, this paper conducts comparative analyses under identical conditions, including consistent training/test splits, preprocessing steps, parameter settings, and adjustment procedures. Additionally, a 5-fold cross-validation strategy with a strict data separation protocol is adopted to ensure that the testing data remain unseen during the model training phase.

### 4.1. Experimental Analysis on Case I: CEC2022 Test Functions

**Figure 4:**
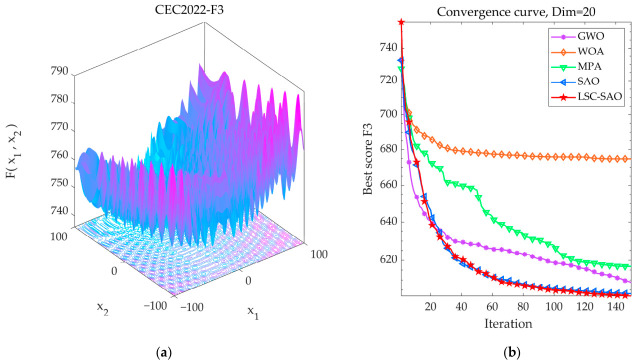
Optimization results. (**a**) Function landscape of F3; (**b**) Convergence curves of optimization algorithms.

**Figure 5:**
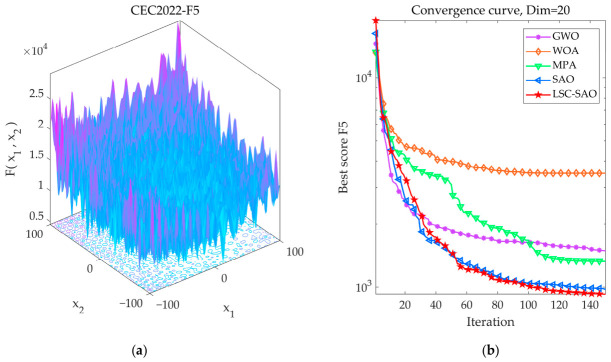
Optimization results. (**a**) Function landscape of F5; (**b**) Convergence curves of optimization algorithms.

**Figure 6:**
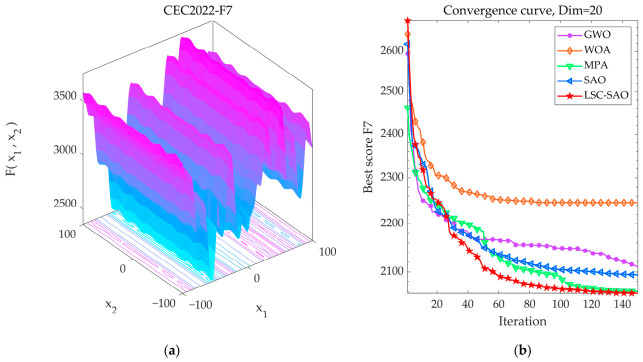
Optimization results. (**a**) Function landscape of F7; (**b**) Convergence curves of optimization algorithms.

**Figure 7:**
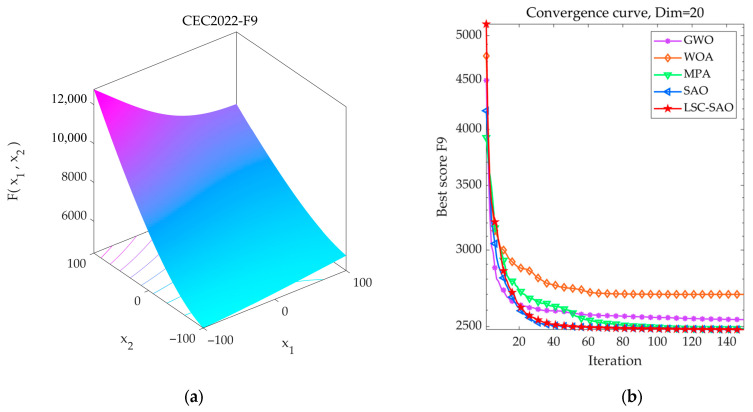
Optimization results. (**a**) Function landscape of F9; (**b**) Convergence curves of optimization algorithms.

**Figure 8:**
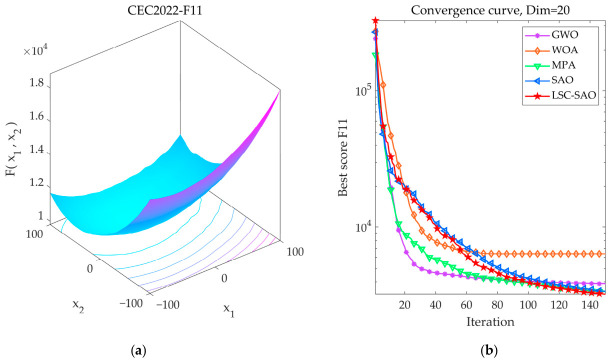
Optimization results. (**a**) Function landscape of F11; (**b**) Convergence curves of optimization algorithms.

**Figure 9:**
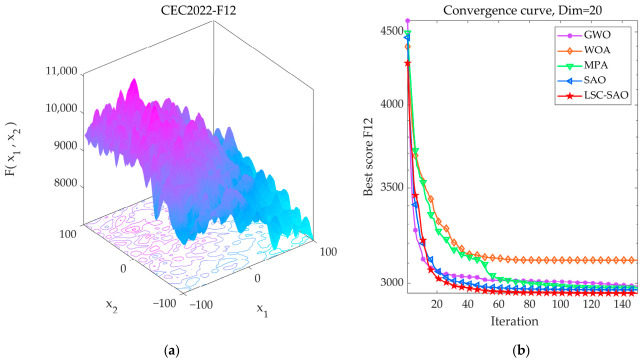
Optimization results. (**a**) Function landscape of F12; (**b**) Convergence curves of optimization algorithms.

**Figure 10:**
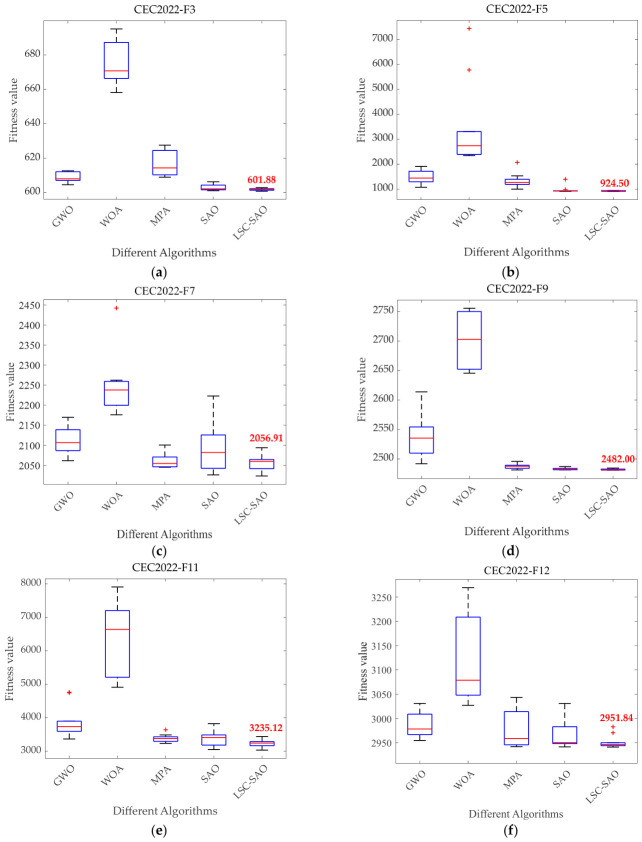
Evaluation of fitness values for various optimization algorithms using CEC2022 test functions. (**a**) Results for F3; (**b**) Results for F5; (**c**) Results for F7; (**d**) Results for F9; (**e**) Results for F11; (**f**) Results for F12.

To preliminarily evaluate the optimization performance of the LSC-SAO algorithm in various complex environments, this study employs the multimodal functions F3 and F5, the hybrid function F7, and the composite functions (including F9, F11, and F12) from the CEC2022 benchmark suite for analysis. First, the parameters of the LSC-SAO are initialized as follows: population size N=30, dimension Dim=20, and maximum number of iterations Max_iter=150. The performance of LSC-SAO is evaluated by repeating the optimization process over 10 independent runs to obtain statistical metrics such as the maximum, minimum, and median fitness values. Simultaneously, the proposed algorithm is compared to GWO, WOA, MPA, SAO, and other approaches. The convergence curves for each function are presented in Figure 4, Figure 5, Figure 6, Figure 7, Figure 8 and Figure 9. Compared to other optimization algorithms, LSC-SAO demonstrates superior convergence speed and stability. This indicates that LSC-SAO exhibits robust performance when dealing with optimization problems of varying complexity, particularly high-complexity functions. The boxplots shown in Figure 10a–f visually illustrate their stability and optimization performance. The LSC-SAO algorithm optimizes the test functions, yielding average fitness values of 601.88, 924.50, 2056.91, 2482.00, 3235.12, and 2951.84, respectively. Furthermore, it maintains optimization stability, with standard deviations of 0.65, 15.46, 21.05, 1.20, 124.87, and 13.68, respectively. As shown in Table 1, metrics such as the worst value, optimal value, and median also demonstrate the advantages of LSC-SAO. Through the detailed comparative experiments mentioned above, it is conclusively demonstrated that LSC-SAO not only outperforms other algorithms but also surpasses the original SAO.

Therefore, LSC-SAO can be used to adaptively select the control parameter of RCMDSE and the key parameters of SVM, thereby enabling more accurate extraction of fault features and improving fault recognition rates.

### 4.2. Experimental Validation on Case II: Planetary Gearbox Dataset

**Figure 11:**
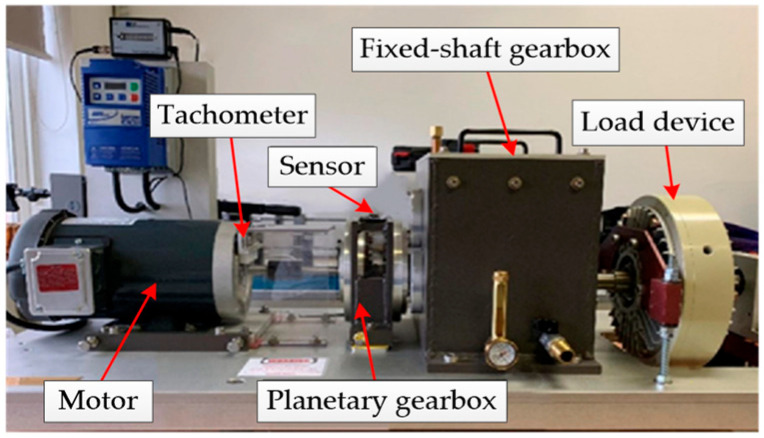
The wind turbine drivetrain test rig in Case II.

**Figure 12:**
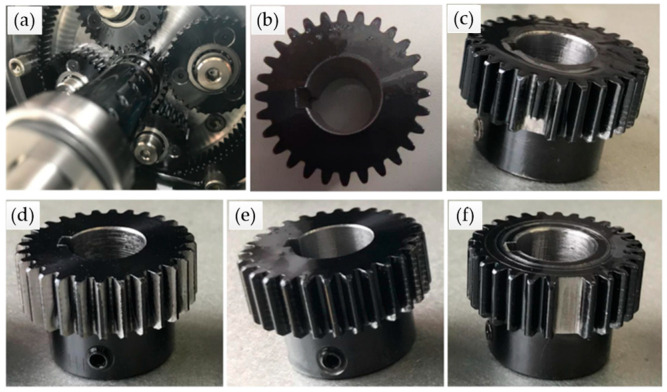
Internal structure of the planetary gearbox and five health conditions in Case II. (**a**) Internal structure of planetary gearbox; (**b**) Healthy condition; (**c**) Gear with a broken tooth; (**d**) Wear gear; (**e**) Crack occurs in the root; (**f**) Missing one tooth.

The fault dataset was collected from the planetary gearbox of a wind turbine drive train test rig, as illustrated in Figure 11. The test rig comprises a drive motor, a planetary gearbox, a fixed-shaft gearbox, and a loading device. The planetary gearbox system comprises four planetary gears rotating around a sun gear, as shown in Figure 12a. The five health conditions of the sun gear are illustrated in Figure 12b–f. Vibration signals were acquired using a Sinocera CA-YD-1181 accelerometer (Sinocera Piezotronics, Inc., Yangzhou, China), and velocity pulses were recorded using an encoder. All channels were sampled at 48 kHz.

In this paper, planetary gearboxes under five different health conditions were analyzed: healthy, broken tooth, wear gear, root crack, and missing tooth. The vibration signals selected for analysis were obtained from the X-axis channel after the second disassembly and reassembly, under an operating speed of 35 Hz. Each sample has a data length of 2048. There are 100 samples for each fault type, resulting in a total of 500 samples across the five fault categories. Table 2 details the relevant information for each sample dataset, including the fault IDs (e.g., B2_35, N2_35, M2_35, R2_35, and W2_35), their corresponding fault labels (1–5, respectively), and the division ratio between the training and testing sets (80% and 20%, respectively). The signal preprocessing steps for this experiment, as well as the parameter settings and adjustment procedures for each algorithm, are detailed in Section 3 of this paper. Given the inherent nonlinearity and non-stationarity of gearbox vibration signals, relying solely on time-domain waveform analysis is insufficient for effectively characterizing their fault features. Therefore, advanced feature extraction and fault classification algorithms must be employed. By effectively constructing a feature space, precise identification and intelligent diagnosis of fault types can be achieved, thereby ensuring the reliability and safety of the gear transmission system operation.

**Figure 13:**
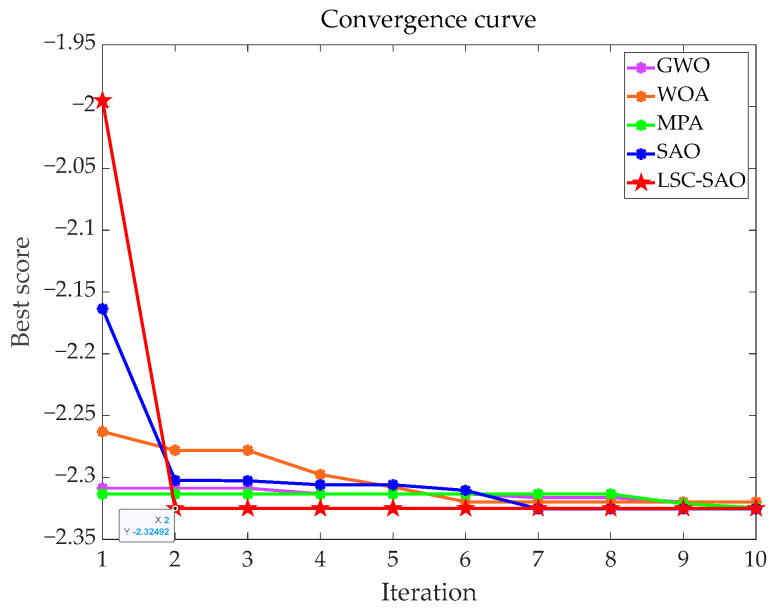
Convergence curve of the fitness value for adaptive optimization of RCMDSE control parameter using LSC-SAO in Case II.

**Figure 14:**
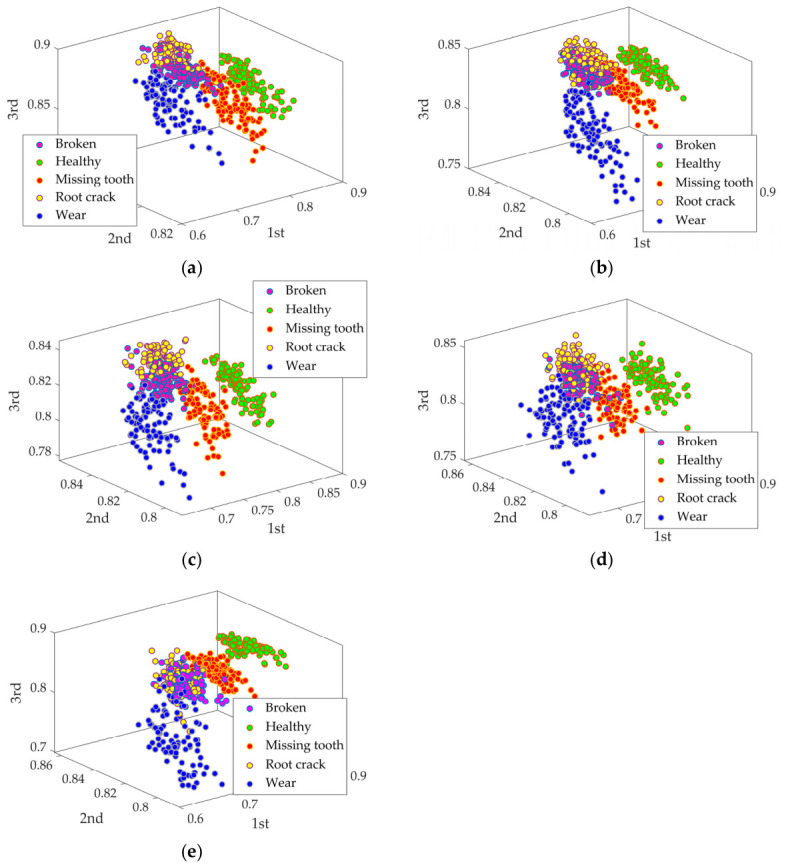
Distribution of the first three feature values of the five adaptive DSEs under five health states in Case II. (**a**) Adaptive-RCMDSE; (**b**) Adaptive-TSMDSE; (**c**) Adaptive-CMDSE; (**d**) Adaptive-MDSE; (**e**) Adaptive-HDSE.

In this study, the adaptive control parameter α of DSE is optimized using the LSC-SAO algorithm. By effectively obtaining $\alpha_{Best}$, the Adaptive-RCMDSE value is calculated as the optimal fault feature of the gearbox. Figure 13 shows the convergence curve of the fitness value $fitness_{\alpha }$. In the second iteration, the value reaches −2.3249 and tends to stabilize. Comparative analysis reveals that LSC-SAO exhibits the fastest convergence rate and the smallest fitness value among the tested algorithms (GWO, WOA, MPA, and SAO), demonstrating its effectiveness in optimizing the control parameter of Adaptive-RCMDSE. In conclusion, the application of LSC-SAO yields a $\alpha_{Best}$ value of 0.6706 for Adaptive-RCMDSE. The distributions of the first three feature values for all fault types under five health states are shown in Figure 14a. With the exception of a few Root crack (Label 4) and Broken (Label 1) samples, the differences in fault features among other categories are significantly more pronounced. This demonstrates that Adaptive-RCMDSE possesses superior feature extraction performance. In order to verify the superiority of the feature extraction method based on Adaptive-RCMDSE in this paper, Adaptive-TSMDSE, Adaptive-CMDSE, Adaptive-MDSE, and Adaptive-HDSE are used for comparative analysis. As shown in Table 3, the values of $\alpha_{Best}$ and $fitness_{\alpha }$ differ, indicating that the LSC-SAO algorithm is effective. The fault feature distributions of the above four methods are illustrated in Figure 14b–e, respectively. It can be seen from the figure that the $fitness_{\alpha }$ value of Adaptive-MDSE is only −1.4139, resulting in insignificant differences among the various fault features. The $fitness_{\alpha }$ value of Adaptive-RCMDSE is −2.3249. However, the fault separability of the other methods is inferior to that of Adaptive-RCMDSE. This indicates that a smaller $fitness_{\alpha }$ value does not necessarily imply superior performance in adaptive fault feature extraction. Compared to Adaptive-HDSE, the operation time of Adaptive-RCMDSE is extended by 31.28 s. Nevertheless, compared to Adaptive-TSMDSE, the operation time of Adaptive-RCMDSE is shortened by 24.65 s. Furthermore, the optimization times for the control parameter of Adaptive-RCMDSE, Adaptive-TSMDSE, Adaptive-CMDSE, Adaptive-MDSE, and Adaptive-HDSE are 3294.67 s, 5764.56 s, 3129.42 s, 469.98 s, and 157.83 s, respectively, indicating high computational costs. Therefore, the feature matrix extracted by Adaptive-RCMDSE is selected for gearbox fault recognition, with a dimension of 5×100×20. However, the feature extraction results of the five adaptive DSEs overlap with different fault states to some extent, making them difficult to distinguish completely.

**Figure 15:**
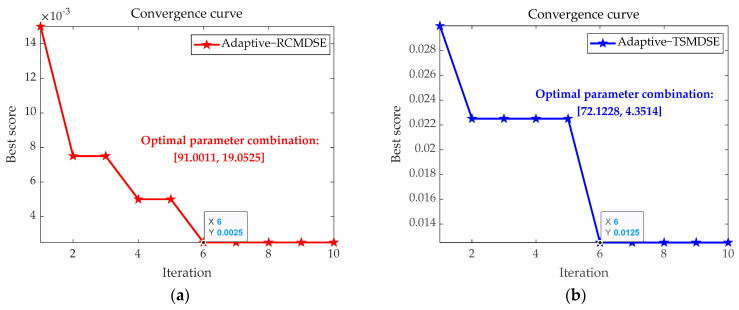

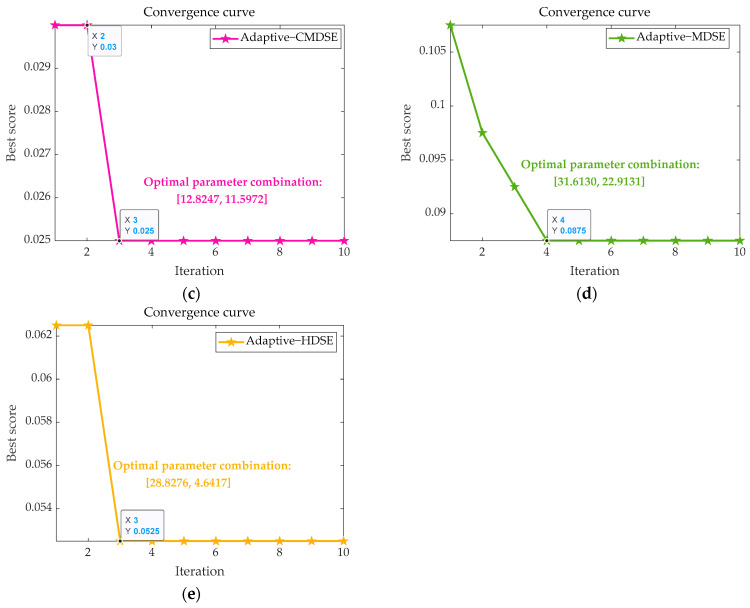
Convergence curves of Adaptive-SVM parameter optimization using LSC-SAO in Case II. (**a**) Adaptive-RCMDSE; (**b**) Adaptive-TSMDSE; (**c**) Adaptive-CMDSE; (**d**) Adaptive-MDSE; (**e**) Adaptive-HDSE.

**Figure 16:**
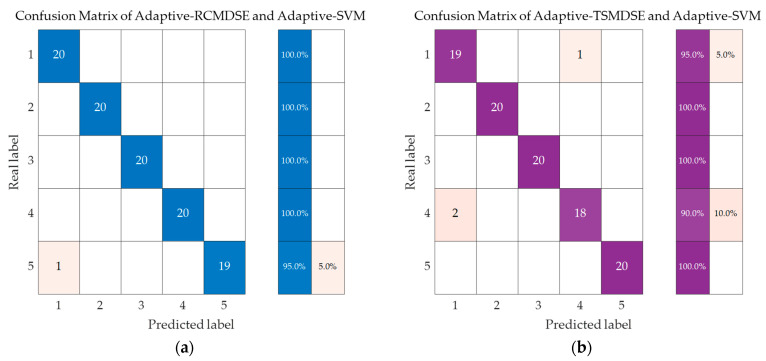

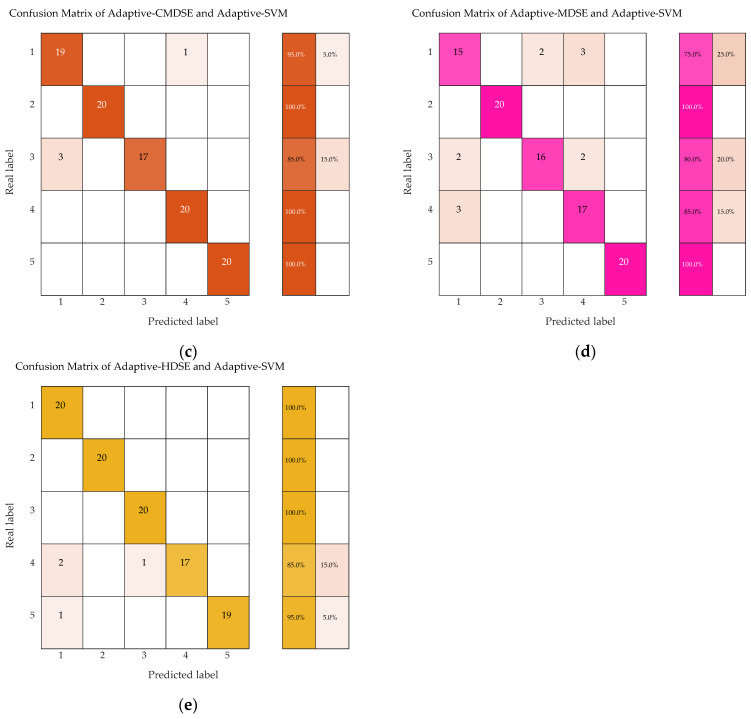
Confusion matrices for fault classification using different DSE feature extraction methods with Adaptive-SVM in Case II. (**a**) Adaptive-RCMDSE; (**b**) Adaptive-TSMDSE; (**c**) Adaptive-CMDSE; (**d**) Adaptive-MDSE; (**e**) Adaptive-HDSE.

In order to fully verify the significant advantages of the Adaptive-RCMDSE algorithm in feature extraction, this paper employs the Adaptive-SVM classification method for accurate fault diagnosis and analysis of the gearbox. First, Adaptive-RCMDSE, Adaptive-TSMDSE, Adaptive-CMDSE, Adaptive-MDSE, and Adaptive-HDSE are used to realize the parameter combination adaptation of the adaptive SVM classification model. The convergence curves and the optimal SVM parameter combinations are shown in Figure 15a–e, respectively. When the iteration reaches the sixth step, the process tends to stabilize. The optimal fitness value of Adaptive-RCMDSE and Adaptive-SVM is 0.0025, and the optimal parameter combination is obtained as [91.0011, 19.0525]. The fault classification accuracy reaches 99.00%. As shown in the confusion matrix in Figure 16a, only one sample (Label 5: Wear) is misclassified (Label 1: Broken). Although the convergence curves of the other four methods converge quickly and tend to stabilize, their fitness values are significantly lower than those of Adaptive-RCMDSE and Adaptive-SVM. Specifically, the values are 0.0125, 0.025, 0.0875, and 0.0525. As shown in Figure 16b–e, 3, 4, 12, and 4 gearbox fault samples are misidentified, respectively. The above comparison demonstrates that Adaptive-RCMDSE more effectively extracts gearbox fault features, enabling accurate classification.

**Figure 17:**
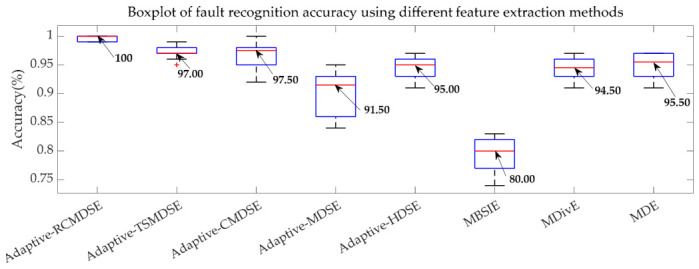
Boxplot of fault classification recognition rates using different entropy features in Case II.

**Figure 18:**
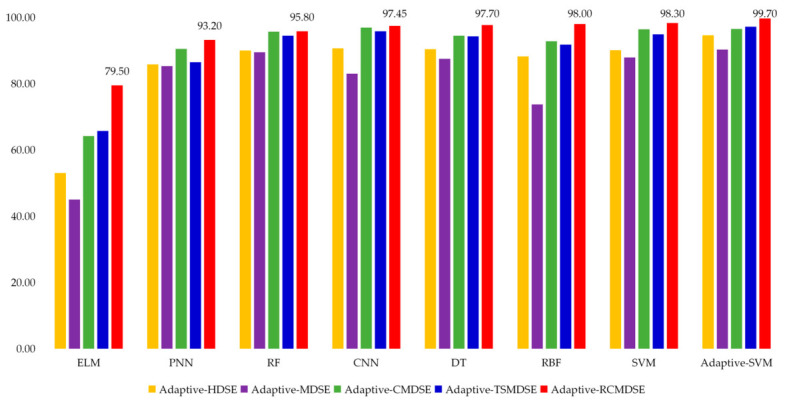
Comparison of average recognition accuracy (%) against other conventional fault classification methods in Case II.

At the same time, the experiment was repeated 10 times to calculate the average results for a more comprehensive and detailed analysis. All experimental comparison results are shown in Figure 17. The average fault classification accuracies for the five different adaptive DSE methods are 99.70%, 97.20%, 96.50%, 90.30%, and 94.60%, respectively. In addition, MBSIE, MDivE, and MDE are introduced for further comparison. The average classification accuracies of these three methods are 79.50%, 94.40%, and 94.70%, respectively, further confirming the superiority of Adaptive-RCMDSE. As shown in Table 4, a more comprehensive comparison is presented. The optimal, worst, standard deviation, median, and mean accuracies of fault classification based on Adaptive-RCMDSE and Adaptive-SVM are 100%, 99.00%, 0.0048, 100%, and 99.70%, respectively. Each index demonstrates superiority over the other seven methods. Furthermore, these results verify that the fault feature extraction capability of DSE outperforms state-of-the-art techniques such as BSIE, DivE, and classic DE. Subsequently, to fully demonstrate the fault classification capability of Adaptive-SVM, this paper also compares and analyzes it against methods, such as ELM, PNN, RF, CNN, DT, RBF, and SVM (with default parameters). The average fault classification accuracies based on different adaptive DSEs and classification models are shown in Figure 18. Among them, when using Adaptive-RCMDSE for feature extraction, the recognition accuracy of Adaptive-SVM is 20.2%, 6.5%, 3.9%, 2.25%, 2%, 1.7%, and 1.4% higher than that of the other commonly used classifiers, respectively. This indicates that Adaptive-SVM can dynamically adapt to changes in operating conditions and continuously learn new features, thereby improving model accuracy. First, learning more essential features (Adaptive-RCMDSE) to deal with unknown samples effectively improves generalizability. Second, it can effectively resist outlier interference and maintain the robustness of the model. When comparing the classification recognition rates of Adaptive-SVM and SVM, Adaptive-SVM outperforms SVM by 1.40%. Adaptive-SVM can dynamically adjust model parameters to adapt to data changes, combining high generalizability and strong robustness. Moreover, compared to the recognition rates of the other four adaptive feature extraction methods, the results of Adaptive-RCMDSE are better, regardless of the classifier used. Compared to SVM, Adaptive-SVM exhibits superior classification performance regardless of the type of adaptive multiscale differential symbolic entropy employed. Therefore, through the comparative analysis of the above experiments, it is confirmed that Adaptive-RCMDSE has good fault characterization ability and Adaptive-SVM has good classification performance.

Finally, this paper introduces GWO, WOA, MPA, and SAO to compare with the LSC-SAO algorithm. Table 5 presents the detailed evaluation indices of the optimal adaptive parameter combination (including the control parameter of RCMDSE and the key parameters of SVM), detailed evaluation metrics for fault classification, and their respective optimization times. Among them, the optimal control parameter of LSC-SAO-RCMDSE is 0.6706, and the optimization values of the other methods are 0.6722, 0.6681, 0.6702, and 0.6708, respectively. The optimal parameter combination for LSC-SAO-SVM is (91.0011, 19.0525), and the detailed evaluation metrics for fault classification (in order of optimal accuracy, worst accuracy, standard deviation, median accuracy, and mean accuracy) are also superior to the those of the other four methods. The above results indicate that the reasonable selection of parameters α and (C, γ) will effectively improve the correlation performance between RCMDSE and SVM. The results in Table 5 indicate that the fault classification metrics of LSC-SAO are 100%, 99%, 0.0048, 100%, and 99.70%, respectively, which are superior to those of SAO (100%, 96%, 0.0148, 99.50%, and 98.80%, respectively). Comparative analysis confirms that LSC significantly enhances the global optimization capability of SAO, effectively preventing the algorithm from falling into local optima. This enables LSC-SAO-RCMDSE to have the optimal fault characterization capability and LSC-SAO-SVM to have the optimal classification performance. Meanwhile, LSC-SAO-RCMDSE and LSC-SAO-SVM have optimization times of 3294.67 s and 29.40 s, respectively, which are also superior to those of the other methods. This indicates that LSC-SAO not only has the best optimization performance but also effectively reduces computational costs. In addition, to verify the sensitivity of the proposed method to different time series lengths, vibration signals with lengths of 2048, 4096, and 8192 are analyzed separately. As shown in Table 6, the mean classification accuracies are 99.70%, 99.70%, and 100%, respectively, indicating good performance. The optimal fitness of LSC-SAO-RCMDSE is −2.3249, −4.8243, and −10.0184, respectively. The optimal fitness of LSC-SAO-SVM is 0.0025, 0.0025, and 0, respectively. However, as the length of the time series increases, the optimization time of $\alpha_{Best}$ will be significantly prolonged, with values of 3294.67 s, 5458.57 s, and 9868.98 s, respectively. Therefore, when using LSC-SAO-RCMDSE and LSC-SAO-SVM for fault diagnosis of vibration signals with a time series length of 2048, all evaluation metrics are optimal.

The Adaptive-RCMDSE algorithm proposed in this study effectively extracts fault features from gearboxes. Furthermore, the classification model based on Adaptive-SVM demonstrates superior fault recognition performance.

### 4.3. Experimental Validation on Case III: Ball Bearing Dataset

**Figure 19:**
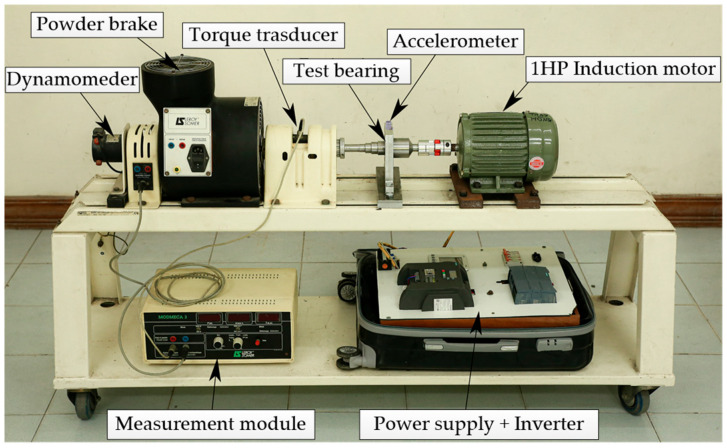
Bearing test bench in Case III.

**Figure 20:**
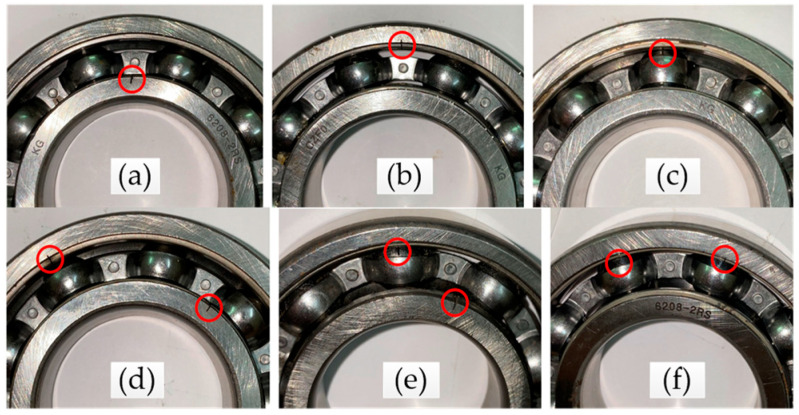
Illustrations of defects on actual test bearings in Case III. (**a**) Inner race crack; (**b**) Outer race crack; (**c**) Ball crack; (**d**) Inner and Outer race cracks; (**e**) Inner race and Ball cracks; (**f**) Outer race and Ball cracks.

To further validate the effectiveness of the method proposed in this paper, classification experiments were conducted on the ball bearing fault diagnosis dataset provided by Hanoi University of Science and Technology in Vietnam. The basic layout of the test rig is shown in Figure 19. The test rig comprises a 750 W (1 HP) induction motor driving a multi-stage shaft and a Leroy Somer powder brake. An inverter controls the motor, and the powder brake serves as a simulated load. The bearings used to introduce defects include KG Bearing India’s 6204, 6205, 6206, 6207, and 6208 ball bearings. The load conditions of the test rig include 0 HP, 200 W, and 400 W, coded as 00, 02, and 04, respectively. The vibration signals selected in this paper were collected from type 6205 ball bearings under 0 HP working conditions, including six types of artificially induced defects and normal bearings. The ball bearing of 6205 has an inner diameter of 25 mm, an outer diameter of 52 mm, a ball diameter of 7.8 mm, and 9 balls. The defects include inner race cracks, outer race cracks, ball cracks, and two combinations thereof, as shown in Figure 20a–f. We followed the same sample collection method as described in Case II. In total, 100 samples were collected, with each sample containing 2048 data points. Subsequently, 80% of the data were randomly selected as training samples, and the remaining 20% were used as test samples. Similarly, the signal preprocessing steps for this experiment, along with the parameter settings and adjustment procedures for each algorithm, are all described in Section 3 of this study. A total of 10 rounds of experiments on ball bearing fault classification were conducted in this paper. The detailed description of the selected data is presented in Table 7, with health condition labels ranging from 1 to 7. The fault diagnosis of the ball bearing is accomplished through subsequent adaptive fault feature extraction and adaptive classification identification.

**Figure 21:**
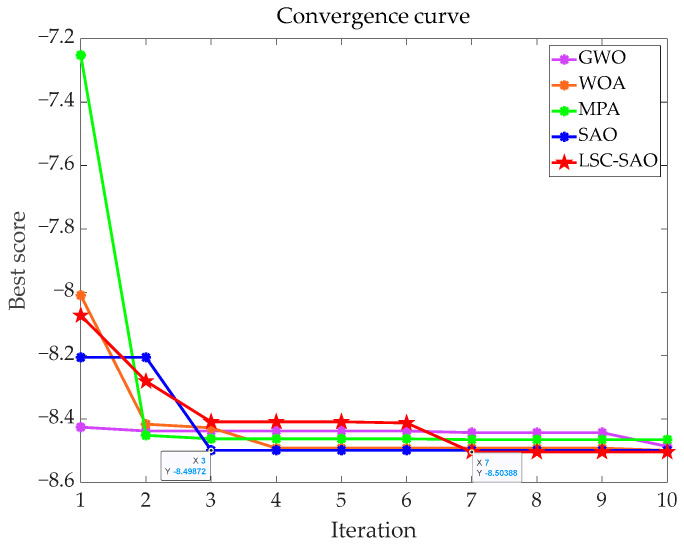
Convergence curve of the fitness value for the adaptive optimization of RCMDSE control parameter using LSC-SAO in Case III.

**Figure 22:**
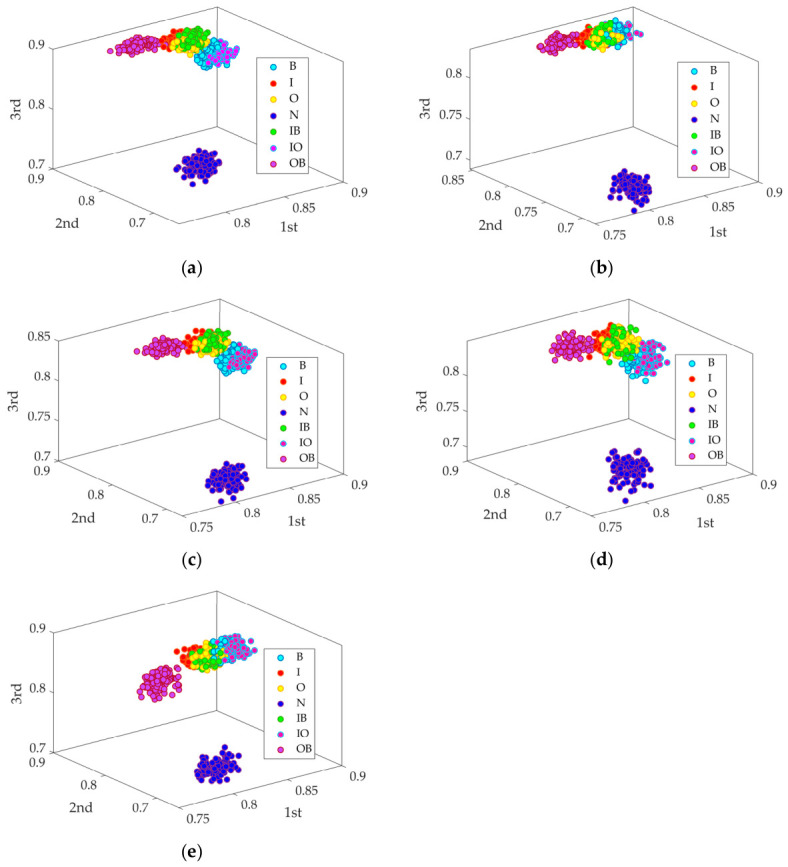
Distribution of the first three feature values of the five adaptive DSEs from ball bearings under seven health states in Case III. (**a**) Adaptive-RCMDSE; (**b**) Adaptive-TSMDSE; (**c**) Adaptive-CMDSE; (**d**) Adaptive-MDSE; (**e**) Adaptive-HDSE.

Similarly, LSC-SAO is used to adaptively select the control parameter α of RCMDSE, TSMDSE, CMDSE, MDSE, and HDSE. The key performance indicators of the above five adaptive feature extraction methods are shown in Table 8. The $\alpha_{Best}$ values are 0.7665, 0.8077, 0.7656, 0.7845, and 0.7813, respectively. The convergence curve based on Adaptive-RCMDSE is shown in Figure 21. When the algorithm iterates to the 7th time, its fitness value reaches −8.5039 and tends to stabilize, which is better than the other four methods. The operation time of Adaptive-RCMDSE is 43.53 s. Compared to the operation time of Adaptive-TSMDSE (83.70 s), the efficiency of Adaptive-RCMDSE is nearly doubled, but its $fitness_{\alpha }$ value is far lower than that of Adaptive-TSMDSE (its fitness value is −32.4416). The operation times of Adaptive-CMDSE and Adaptive-RCMDSE are relatively close. However, due to their computational complexity, the calculation times of Adaptive-MDSE and Adaptive-HDSE are only 6.70 s and 2.23 s, respectively. Figure 22a–e shows the distribution of the first three fault feature values of ball bearings under seven different health conditions. As shown in Figure 22a, the clustering degree of fault features of each category is distinct, which indicates that the feature extraction performance of the Adaptive-RCMDSE method is effective. The $fitness_{\alpha }$ values based on the Adaptive-MDSE and Adaptive-HDSE methods are −3.0804 and −12.3886, respectively. The distribution of fault features is shown in Figure 22d,e. Although the operation times of these two methods are relatively short, the distinction between different types is not obvious, which easily leads to low efficiency of fault identification. This is also consistent with the experimental results in Section 4.2. A smaller $fitness_{\alpha }$ value does not necessarily reflect a more significant adaptive fault feature extraction method. For this reason, this paper continued to select Adaptive-RCMDSE as the fault feature extraction method for ball bearing fault recognition, and the size of its feature matrix is 7×100×20.

**Figure 23:**
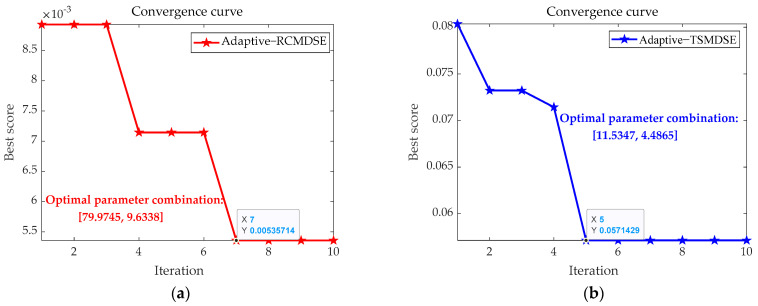

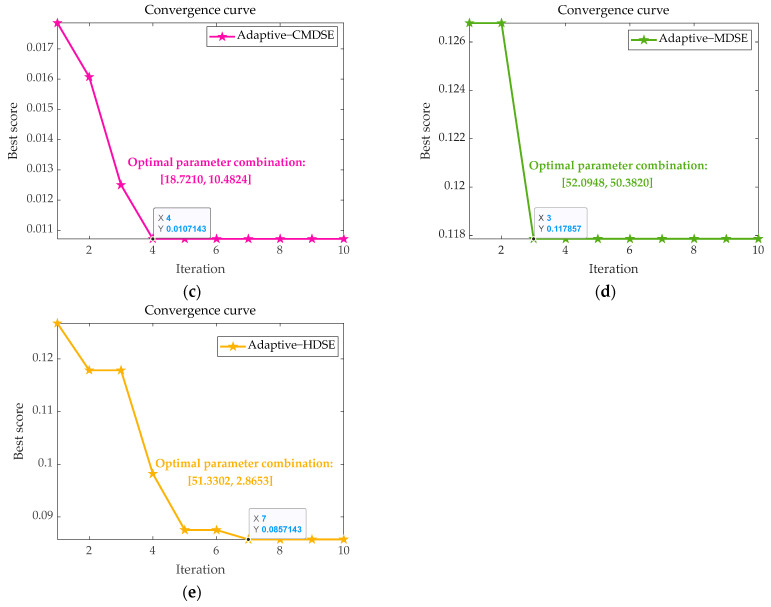
Convergence curves of Adaptive-SVM parameter optimization using LSC-SAO in Case III. (**a**) Adaptive-RCMDSE; (**b**) Adaptive-TSMDSE; (**c**) Adaptive-CMDSE; (**d**) Adaptive-MDSE; (**e**) Adaptive-HDSE.

**Figure 24:**
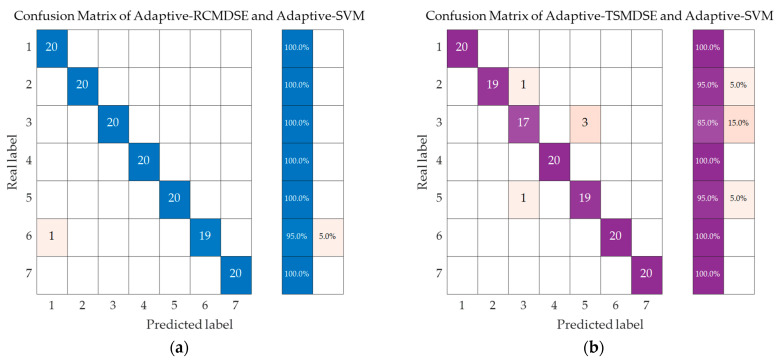

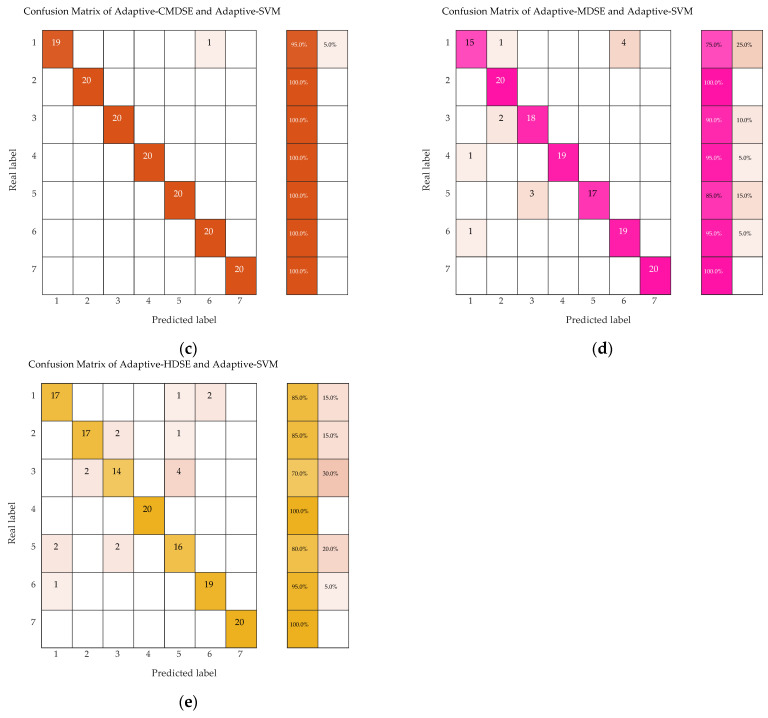
Confusion matrices for fault classification using different DSE feature extraction methods with Adaptive-SVM in Case III. (**a**) Adaptive-RCMDSE; (**b**) Adaptive-TSMDSE; (**c**) Adaptive-CMDSE; (**d**) Adaptive-MDSE; (**e**) Adaptive-HDSE.

The Adaptive-SVM classification method is used again to diagnose faults in ball bearings. First, five kinds of adaptive DSEs are used to determine the adaptive parameter combination of the SVM classification model. The combination of their convergence curves and optimal SVM parameters is shown in Figure 23a–e, respectively. The convergence curves of the SVM fitness value tend to stabilize at the 7th, 5th, 4th, 3rd, and 7th iterations, with values of 0.005357, 0.05714, 0.01071, 0.11786, and 0.0857, respectively. The optimal parameter combinations of Adaptive-SVM are [79.9745, 9.6338], [11.5347, 4.4865], [18.7210, 10.4824], [52.0948, 50.3820], and [51.3302, 2.8653], respectively. It can be concluded that the smallest fitness value is obtained by training Adaptive-SVM using Adaptive-RCMDSE as the fault feature of the ball bearing. Then, classification tests were carried out, and the confusion matrices of the results are shown in Figure 24a–e. As shown in Figure 24a, the classification accuracy of Adaptive-RCMDSE combined with Adaptive-SVM reaches 99.29%, and only one sample (Label 6: IO) is misclassified (Label 1: B). Using the four other methods for diagnosis, 5, 1, 12, and 17 samples of ball bearing faults are misclassified, with recognition rates of 96.43%, 99.29%, 91.43%, and 87.86%, respectively. The above comparison also demonstrates that Adaptive-RCMDSE can better adaptively characterize the fault features of ball bearings, thereby achieving accurate classification.

**Figure 25:**
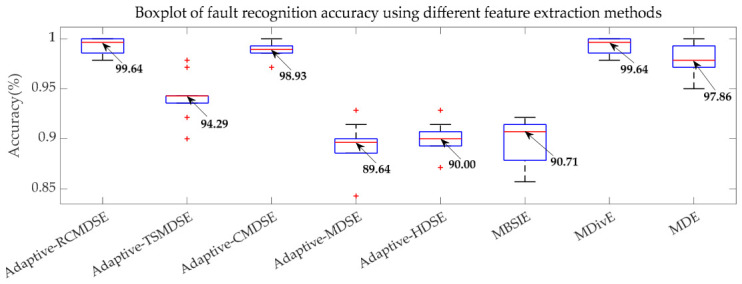
Boxplot of fault classification recognition rates using different entropy features in Case III.

**Figure 26:**
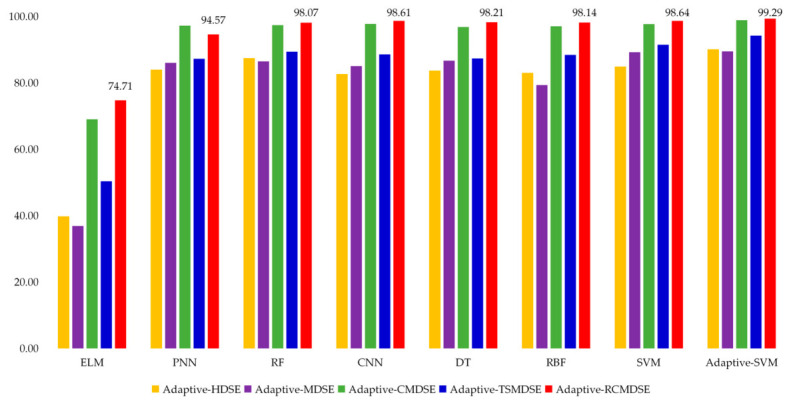
Comparison of average recognition accuracy (%) against other conventional fault classification methods in Case III.

This paper repeated the above experiment 10 times and calculated the average of their classification results. The comparison results of all experiments are shown in Figure 25. The average fault classification accuracies for the five different adaptive DSEs, MBSIE, MDivE, and MDE are 99.29%, 94.21%, 98.86%, 89.43%, 90.07%, 89.50%, 99.29%, and 97.86%, respectively. Adaptive-RCMDSE yields the best recognition performance. Through the detailed comparative experiments mentioned above, it is further demonstrated that LSC-SAO has significant advantages in adaptive adjustment of RCMDSE control parameter. When Adaptive-RCMDSE serves as the fault feature for ball bearings and is classified by Adaptive-SVM, the optimal classification accuracy is 100%, the worst accuracy is 97.86%, the standard deviation is 0.0082, and the median accuracy is 99.64%. All of the above indicators are also superior to (or equivalent to) those of the other seven feature extraction methods, as shown in Table 9 for detailed comparison. In addition, this paper uses five different adaptive DSEs as fault features and employs ELM, PNN, RF, CNN, DT, RBF, and SVM (with default parameters) for fault recognition to conduct a comparative analysis. The obtained results are largely consistent with the experimental results of the BJTU datasets, as shown in Figure 26. Notably, when Adaptive-RCMDSE is used as the fault feature, the classification accuracy of Adaptive-SVM is higher than that of other commonly used classifiers by 24.58%, 4.72%, 1.22%, 0.68%, 1.08%, 1.15%, and 0.65%, respectively. Most significantly, Adaptive-SVM outperforms the standard SVM by 0.65%. This demonstrates that Adaptive-SVM can dynamically adapt to changes in operating conditions and continuously learn new features, thereby improving model accuracy. Furthermore, it proves that the method proposed in this paper can not only improve the generalizability of the model but also maintain its robustness. Similarly, a comparison of the classification recognition rates using the other four adaptive feature extraction methods verifies that the fault characterization capability of Adaptive-RCMDSE is superior to that of the other methods. Comparing Adaptive-SVM with SVM again, regardless of the adaptive multiscale differential symbolic entropy used, Adaptive-SVM still exhibits better classification performance.

Finally, to further demonstrate the effectiveness of LSC-SAO, a comparative analysis is conducted with methods such as GWO, WOA, MPA, and SAO. The optimal adaptive parameter combination of RCMDSE and SVM, detailed evaluation metrics for fault classification, and their respective optimization times are shown in Table 10. The optimal control parameter for Adaptive-RCMDSE obtained in this experiment is 0.7665, and the optimal parameter combination for Adaptive-SVM is (79.9745, 9.6338). Similarly, the fault classification metrics of this method are superior to those of the other comparative methods. This further demonstrates that employing the LSC-SAO algorithm for the adaptive optimization of RCMDSE and SVM parameters not only effectively extracts and characterizes fault features but also significantly enhances the performance and diagnostic accuracy of the classifier. Table 10 also shows that the fault classification metrics of LSC-SAO are 100%, 97.86%, 0.0082, 99.64%, and 99.29%, respectively, which are superior to those of SAO (100%, 97.86%, 0.0082, 99.29%, and 99.29%, respectively). A comparative analysis between LSC-SAO and SAO demonstrates that the LSC strategy effectively enhances the convergence performance and population diversity, thereby successfully circumventing the susceptibility of SAO to local optima. In terms of parameter optimization efficiency, the computational times of Adaptive-RCMDSE and Adaptive-SVM are 4369.8 s and 56.01 s, respectively, which are also shorter than those of the other comparison methods. In this paper, vibration signals with time series lengths of 2048, 4096, and 8192 are analyzed to verify the robustness of the proposed method against varying data lengths, as shown in Table 11. For these three signal lengths, the proposed method achieves high classification accuracy, with average accuracies of 99.29%, 100%, and 100%, respectively. The corresponding optimal fitness values of Adaptive-RCMDSE are −8.5039, −16.8937, and −30.9154, respectively. However, with an increase in time series length, the computational cost of the optimization process rises significantly, and the optimization times extend to 4369.79 s, 7655.32 s, and 13,782.22 s, respectively. Therefore, when vibration signals with lengths of 2048 are employed for analysis, the proposed method outperforms the alternatives in all evaluation metrics, demonstrating excellent comprehensive diagnostic performance.

The comprehensive experimental comparison results confirm that the proposed diagnostic method integrating Adaptive-RCMDSE and Adaptive-SVM possesses significant advantages in terms of accuracy, generalizability, and robustness.

### 4.4. Experimental Validation on Case IV: Axle Box Bearing Dataset

**Figure 27:**
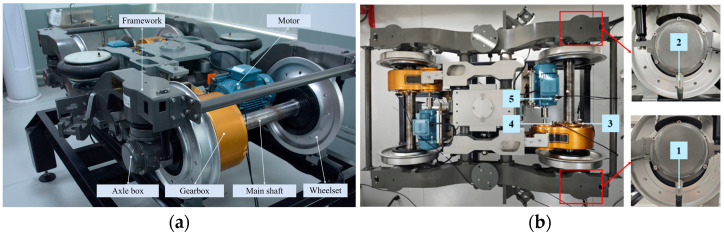
The HTBF test bench: (**a**) Fault simulation platform; (**b**) Sensor layout.

The analysis utilized the HTBF dataset from SWJTU. The vibration signals were acquired from a fault simulation platform designed as a 1:2 scale model of a high-speed train bogie, as shown in Figure 27a. The test bench primarily comprises a framework, motor, gearbox, main shaft, and axle box. It was specifically configured to simulate faults in the axle box and gearbox while allowing for the variation of motor speed and vertical load to replicate diverse operating conditions. The investigation focuses on vibration signals under specific test parameters: a vertical load of 1300 kg (Case V1), a lateral load of 0 kg (Case H1), and a motor speed of 40 Hz (Case S2). For data acquisition, the test bench was instrumented with four MS101050 uniaxial accelerometers (Safran Colibrys/Safran Sensing Technologies, Bevaix, Switzerland) and one TES001T triaxial accelerometer (CMS-One Tech, Changxing, Huzhou, China). Signals from three measurement points (the axle box, gearbox, and motor) were acquired across five channels at a sampling frequency of 25.6 kHz, with the sensor layout detailed in Figure 27b. Finally, this study investigated various axle box bearing fault types utilizing Channel 1 signals.

**Figure 28:**
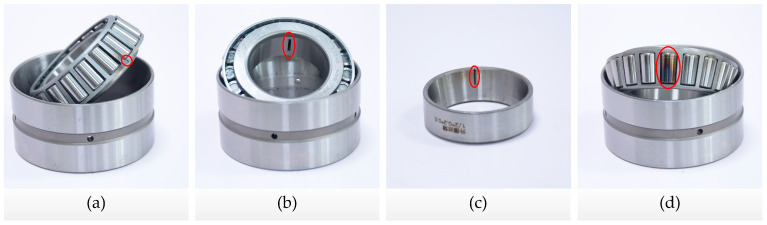
Photographs of four typical faulty bearing components: (**a**) Cage fault: (**b**) Inner race fault; (**c**) Outer race fault; (**d**) Rolling element fault.

The axle box bearing incorporates four simulated fault types: cage fault, inner race fault, outer race fault, and rolling element fault, as depicted in Figure 28. The experimental data characterizing the nine health states of the axle box bearings are summarized in Table 12. Notably, the outer race and rolling element faults comprise three distinct severity levels.

**Figure 29:**
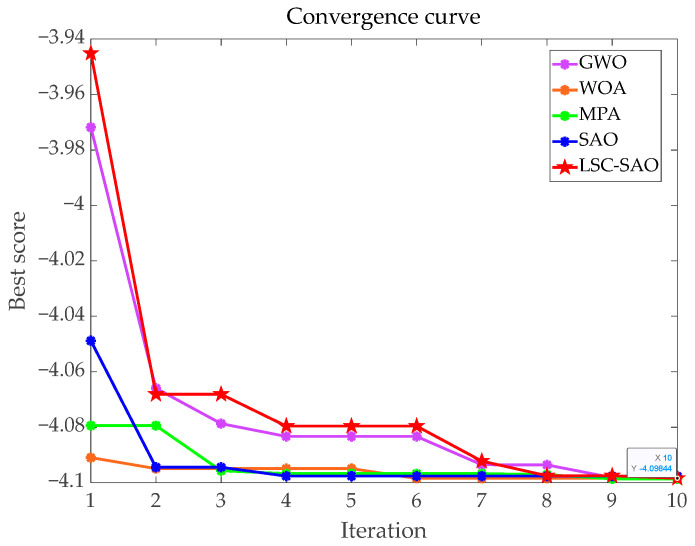
Convergence curve of the fitness value for the adaptive optimization of RCMDSE control parameter using LSC-SAO in Case IV.

**Figure 30:**
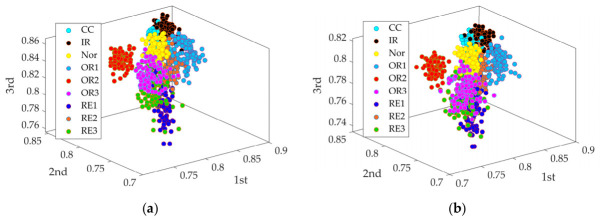

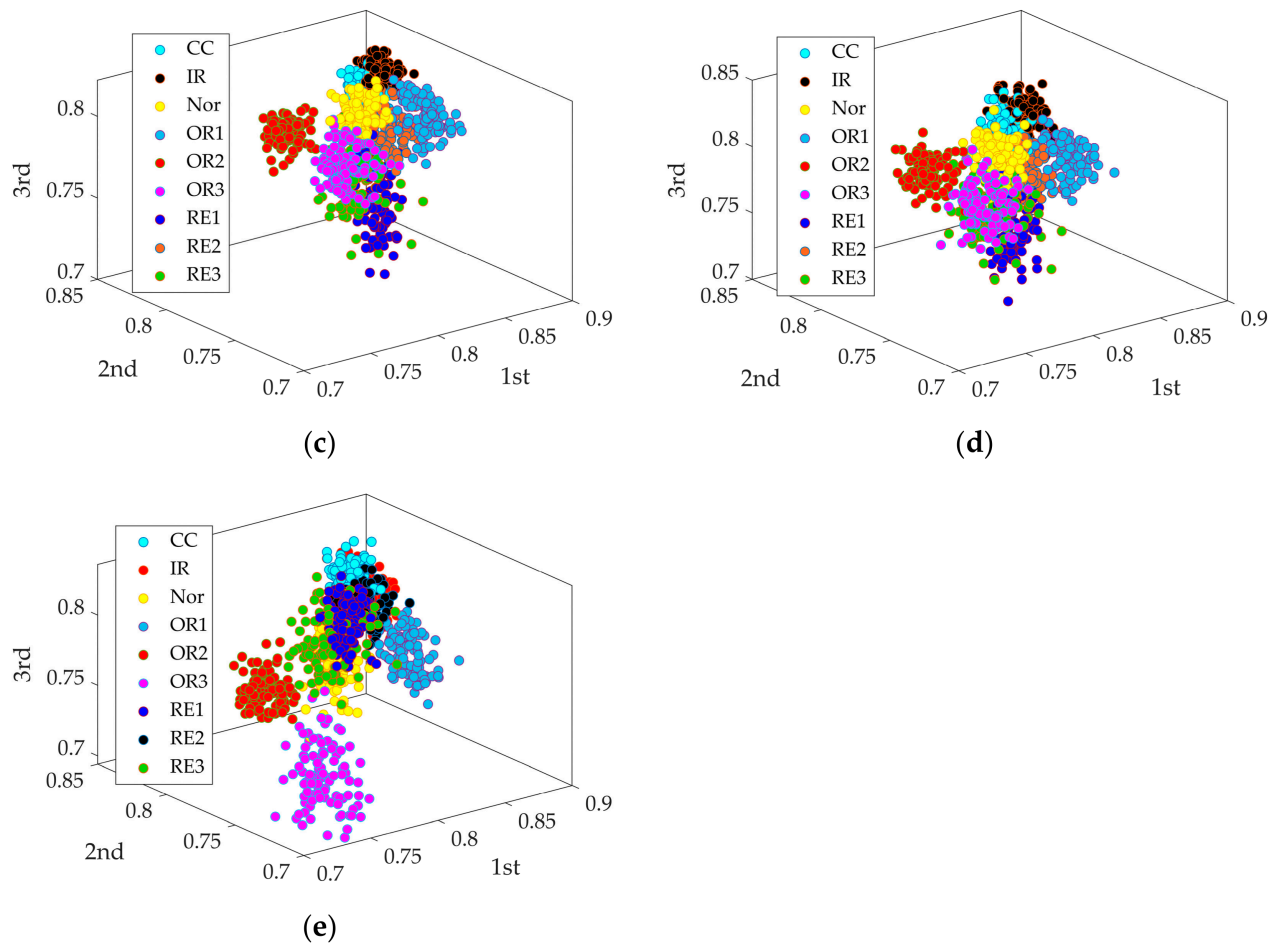
Distribution of the first three feature values of the five adaptive DSEs from axle box bearings under nine health states in Case IV. (**a**) Adaptive-RCMDSE; (**b**) Adaptive-TSMDSE; (**c**) Adaptive-CMDSE; (**d**) Adaptive-MDSE; (**e**) Adaptive-HDSE.

Similarly, LSC-SAO was employed to optimize the control parameter of DSE for extracting fault features from the axle box bearings. Table 13 summarizes the key performance indicators of the five adaptive DSEs. The optimized parameter $\alpha_{Best}$ values are 0.7482, 0.7767, 0.7407, 0.8135, and 0.8402, respectively. Specifically, Adaptive-RCMDSE achieves an optimal fitness value of −4.0984, with an operation time of 59.69 s and an optimization time of 5923.64 s. These results align well with the findings in Cases II and III. As illustrated in Figure 29, which presents the convergence curves of the five optimization algorithms, all curves tend to stabilize after the 7th iteration, with LSC-SAO demonstrating a particularly prominent convergence rate. Figure 30a–e depict the feature distributions corresponding to the nine health states of the axle box bearings. Although Figure 30 exhibits distinct clustering among different fault categories, it is important to note that this visualization reflects only the first three feature values. This study applied minimum preprocessing to maintain the originality of the fault feature distribution, ensuring that the diagnostic results reflect the true performance under actual conditions. Although the distribution patterns are consistent with those in Section 4.2 and Section 4.3, further classification using a high-performance classifier remains necessary. To this end, a feature matrix with dimensions of 9×100×20 is constructed, followed by fault analysis using Adaptive-RCMDSE and Adaptive-SVM.

**Figure 31:**
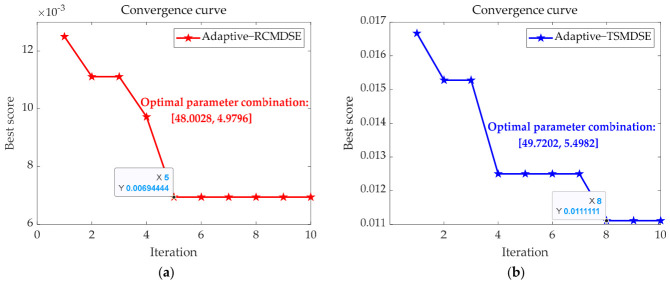

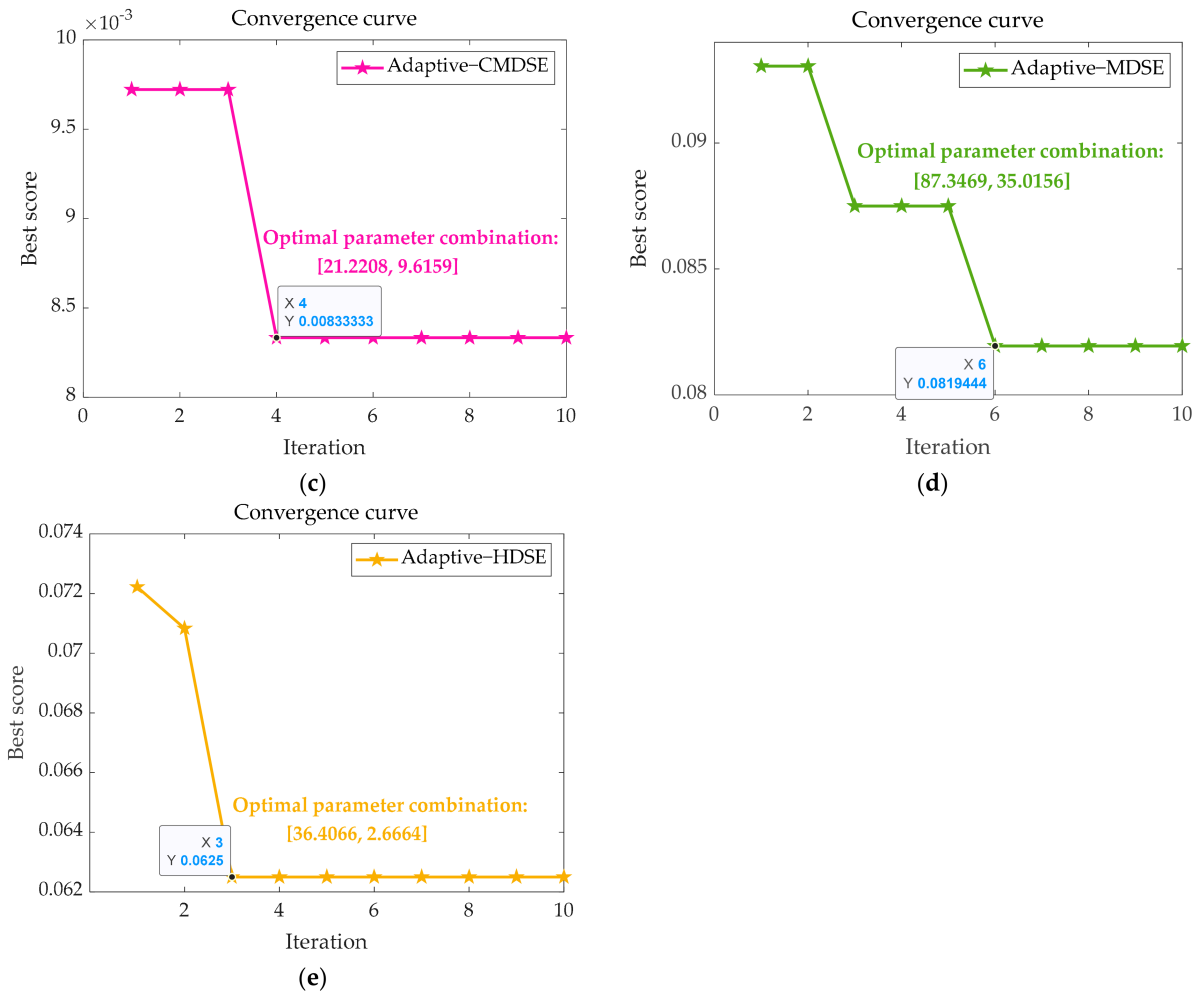
Convergence curves of Adaptive-SVM parameter optimization using LSC-SAO in Case IV. (**a**) Adaptive-RCMDSE; (**b**) Adaptive-TSMDSE; (**c**) Adaptive-CMDSE; (**d**) Adaptive-MDSE; (**e**) Adaptive-HDSE.

**Figure 32:**
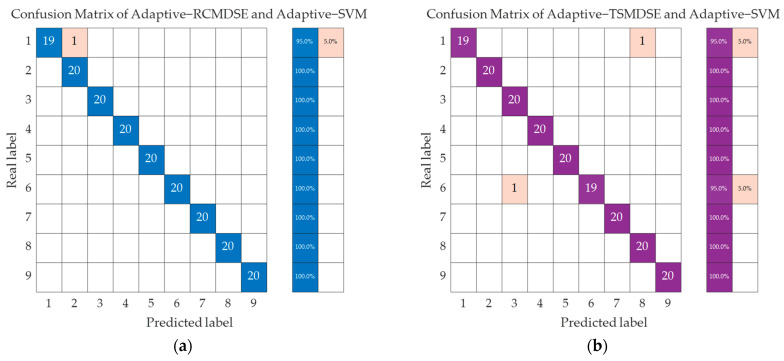

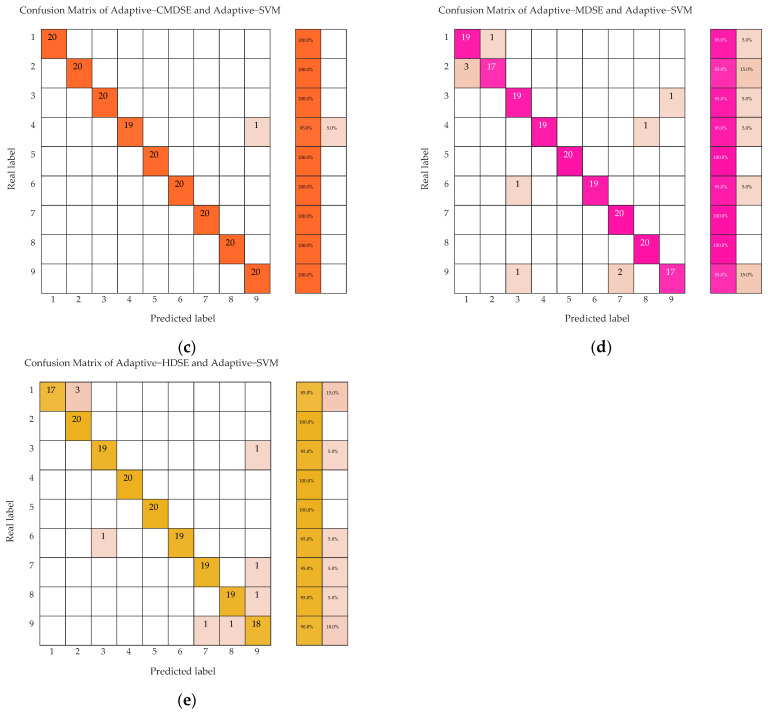
Confusion matrices for fault classification using different DSE feature extraction methods with Adaptive-SVM in Case IV. (**a**) Adaptive-RCMDSE; (**b**) Adaptive-TSMDSE; (**c**) Adaptive-CMDSE; (**d**) Adaptive-MDSE; (**e**) Adaptive-HDSE.

In this section, the Adaptive-SVM was trained using five adaptive DSEs to optimize its key parameters ($C_{Best}$ and $\gamma_{Best}$). Figure 31a–e illustrates the convergence curves of LSC-SAO and the corresponding optimal parameter combinations for the SVM. The fitness values reach their optima at the 5th, 8th, 4th, 6th, and 3rd iterations, respectively, after which the convergence curves tend to stabilize. LSC-SAO yields fitness values of 0.006944, 0.01111, 0.008333, 0.08194, and 0.0625, respectively. Accordingly, the optimal parameter combinations for the Adaptive-SVM are determined as [48.0028, 4.9796], [49.7202, 5.4982], [21.2208, 9.6159], [87.3469, 35.0156], and [36.4066, 2.6664]. With Adaptive-RCMDSE employed as the input feature for training, the experimental results indicate that LSC-SAO achieves the minimum fitness value. The confusion matrices for the Adaptive-SVM classification results are presented in Figure 32a–e. As shown in Figure 32a, the proposed method achieves a classification accuracy as high as 99.44%, with only one sample (Label 1) misclassified as Label 2. By contrast, the other four methods result in 2, 1, 10, and 9 misclassified samples, corresponding to recognition rates of 98.89%, 99.44%, 94.44%, and 95.00%, respectively. The comparative analysis verifies that Adaptive-RCMDSE possesses superior fault representation capabilities and achieves effective adaptive representation.

**Figure 33:**
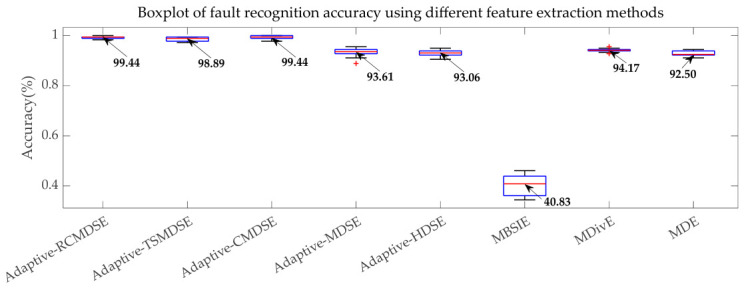
Boxplot of fault classification recognition rates using different entropy features in Case IV.

**Figure 34:**
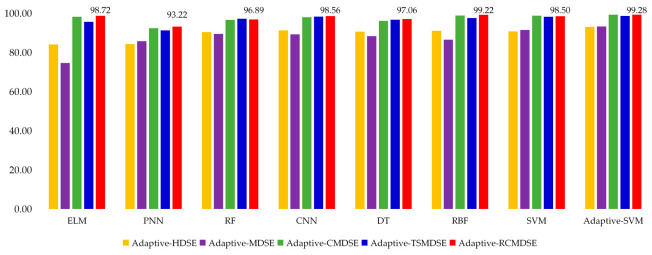
Comparison of average recognition accuracy (%) among different fault classification methods in Case IV.

Subsequently, the training and testing experiments for Adaptive-SVM were repeated 10 times, and the mean results were utilized for comparative analysis. Figure 33 illustrates the box plots of the fault classification recognition rates for the five adaptive DSEs, alongside MBSIE, MDivE, and MDE. The corresponding average accuracies are 99.28%, 98.67%, 99.28%, 93.28%, 93.06%, 40.28%, 94.17%, and 92.83%, respectively. Notably, Adaptive-RCMDSE demonstrates the most superior and stable recognition performance. By employing LSC-SAO to adaptively determine the optimal control parameter for RCMDSE, accurate extraction of fault features from axle box bearings was achieved. Consequently, the Adaptive-SVM classification yields a maximum accuracy of 100% and a minimum of 98.33%, with a standard deviation of 0.0053 and a median of 99.44%. As detailed in Table 14, these results align well with the experimental findings in Section 4.2 and Section 4.3, thereby verifying the superior generalizability of the proposed method. Furthermore, Adaptive-SVM was benchmarked against classifiers including ELM, PNN, RF, CNN, DT, RBF, and SVM (with default parameters) based on feature sets constructed from different DSEs. The results, shown in Figure 34, are consistent with the analysis of the BJTU and HUST datasets. Especially when utilizing Adaptive-RCMDSE as the fault feature, the accuracy of Adaptive-SVM improved by 0.56%, 6.06%, 2.39%, 0.72%, 2.22%, 0.06%, and 0.78% compared to the other classifiers, respectively. This dual adaptive mechanism ensures both optimal fault feature extraction and superior classifier performance.

Table 15 presents the comparative results against the unimproved optimization algorithm. The optimal parameters for Adaptive-RCMDSE and Adaptive-SVM are determined to be 0.7482 and (48.0028, 4.9796), respectively, with all classification metrics surpassing those of the other methods. This comparison clearly demonstrates that the introduction of LSC effectively prevents SAO from becoming trapped in local optima. In terms of parameter optimization efficiency, the proposed method also exhibits superior performance. Furthermore, under varying time series lengths, the method maintains high classification accuracy, achieving average accuracies of 99.28%, 99.83%, and 100%, with corresponding optimal fitness values of −4.0984, −8.0729, and −15.1647. However, it is noteworthy that increasing the time series length significantly raises the computational cost, with optimization times reaching 5923.64 s, 10,330.88 s, and 17,958.79 s, respectively. Detailed results are provided in Table 16.

In summary, the experimental results comprehensively validate the superiority of the proposed method in terms of accuracy, generalizability, and robustness.

## 5. Conclusions

This paper proposes a novel fault diagnosis method based on Adaptive-RCMDSE feature extraction and Adaptive-SVM. This method leverages the advantages of LSC-SAO for the adaptive selection of RCMDSE control parameter and SVM key parameters and performs classification and identification across three datasets: a planetary gearbox, ball bearings, and axle box bearings. The experimental results suggest that the proposed method demonstrates improvements compared to conventional approaches, thereby providing evidence for its effectiveness and innovative aspects. Furthermore, compared to deep learning methods, it requires fewer feature samples to achieve accurate fault diagnosis.

The main conclusions are as follows:

1. The proposed LSC-improved SAO algorithm achieves rapid and accurate optimization for functions F3, F5, F7, F8, F11, and F12 from the CEC2022, with average fitness values of 601.877, 924.496, 2056.914, 2481.998, 3235.122, and 2951.836, respectively. Simultaneously, it maintains optimized stability, with standard deviations of 0.654, 15.455, 21.049, 1.199, 124.866, and 13.676, respectively. A detailed comparison confirmed that LSC effectively prevents the SAO algorithm from becoming trapped in local optima, thereby enhancing its optimization performance.
2. A fault feature extraction method based on Adaptive-RCMDSE is proposed. Firstly, by improving the computational strategy, the stability and accuracy of DSE are significantly enhanced. Secondly, the method utilizes the within-class and between-class scatter matrices of fault features as the fitness function to achieve adaptive selection of the control parameter α. By adaptively adjusting the control parameter of differential symbolic transformation through LSC-SAO, nonlinear symbolic dynamics are effectively extracted. The experimental results show that the optimal control parameter $\alpha_{Best}$ values for Adaptive-RCMDSE are 0.6706, 0.7665, and 0.7482, respectively, and the optimal fitness values are −2.3249, −8.5039, and −4.0984, respectively. Furthermore, this method outperforms Adaptive-TSMDSE, Adaptive-CMDSE, Adaptive-MDSE, Adaptive-HDSE, and other multiscale entropies, including MBSIE, MDivE, and MDE, in terms of feature extraction quality.
3. The proposed Adaptive-SVM classification method employs 5-fold cross-validation classification accuracy as the fitness function for selecting the penalty factor $C_{Best}$ and kernel parameter $\gamma_{Best}$, thereby constructing an adaptive SVM model. Through fault diagnosis experiments on a gearbox and ball bearings, the optimal SVM parameter combinations obtained by LSC-SAO are [91.0011, 19.0525], [79.9745, 9.6338], and [48.0028, 4.9706], with average recognition rates of 99.70%, 99.29%, and 99.28%, respectively. Furthermore, compared to the other four classification methods (including GWO, WOA, MPA, and SAO), Adaptive-SVM demonstrates superior performance in terms of worst value, standard deviation, median, and mean classification accuracy.

Nevertheless, a limitation of the methodology presented herein is the considerable time required for parameter optimization. Additionally, when processing vibration signals under strong noise conditions, it is essential to pre-process the data using effective denoising methods to eliminate signal components irrelevant to fault classification, thereby ensuring the stability of Adaptive-RCMDSE. The generalizability of the proposed method needs further validation on actual industrial machinery data, which often involves more complex fault modes and severe data imbalance. In future work, we plan to collect real-world vibration signals from on-site machinery to further test the method’s robustness. Additionally, integrating the proposed feature extraction method with transfer learning architectures could be a promising direction to enhance its cross-domain generalizability.

## Figures and Tables

**Table 1:** Evaluation metrics for the fitness values of CEC2022 function.

Function Name	Optimization Algorithm	Optimal Value	Worst Value	Standard Deviation	Median Value	Mean Value
F3	LSC-SAO	600.80	602.89	0.65	601.88	601.88
	SAO	601.10	606.29	1.92	602.18	603.07
	MPA	608.99	627.59	7.47	614.37	616.89
	WOA	658.13	695.08	12.32	670.74	674.71
	GWO	604.59	612.69	2.78	608.00	608.72
F5	LSC-SAO	902.66	943.98	15.46	928.21	924.50
	SAO	905.27	1392.45	148.59	923.80	973.54
	MPA	995.04	2064.97	297.57	1261.57	1330.74
	WOA	2340.10	7428.30	1711.98	2735.51	3499.45
	GWO	1072.06	1906.89	262.77	1440.58	1491.14
F7	LSC-SAO	2023.50	2093.83	21.05	2059.91	2056.91
	SAO	2026.16	2222.84	61.91	2082.02	2094.16
	MPA	2045.21	2100.61	18.19	2054.73	2061.05
	WOA	2175.99	2442.13	76.00	2237.91	2245.41
	GWO	2061.54	2169.69	33.93	2106.61	2110.87
F9	LSC-SAO	2480.99	2484.55	1.20	2481.35	2482.00
	SAO	2481.32	2487.18	2.18	2482.41	2483.35
	MPA	2481.36	2495.83	4.41	2487.41	2487.66
	WOA	2645.42	2755.56	46.08	2702.64	2699.16
	GWO	2491.87	2613.76	40.69	2535.36	2541.75
F11	LSC-SAO	3030.83	3436.10	124.87	3238.55	3235.12
	SAO	3045.84	3820.64	256.64	3402.68	3394.55
	MPA	3231.80	3637.59	118.09	3376.89	3385.13
	WOA	4908.63	7905.38	1048.84	6636.13	6383.84
	GWO	3364.22	4758.69	489.53	3730.50	3871.11
F12	LSC-SAO	2941.17	2982.86	13.68	2946.25	2951.84
	SAO	2941.58	3030.88	34.61	2950.17	2968.71
	MPA	2941.97	3043.37	40.96	2958.84	2977.77
	WOA	3027.11	3269.23	87.24	3078.67	3114.52
	GWO	2954.69	3030.95	26.52	2978.29	2985.64

**Table 2:** Internal structure of the planetary gearbox and five health conditions in Case II.

Healthy Condition	Fault ID	Label	Time Series Length	Number of Samples	Train Set Ratio	Test Set Ratio
Broken	B2_35	1	2048	100	0.8	0.2
Healthy	N2_35	2	2048	100	0.8	0.2
Missing tooth	M2_35	3	2048	100	0.8	0.2
Root crack	R2_35	4	2048	100	0.8	0.2
Worn	W2_35	5	2048	100	0.8	0.2

**Table 3:** Key performance indicators of the fault feature extraction method using DSEs with adaptive control parameters in Case II.

Feature Extraction Method	$\alpha_{Best}$	Best $fitness_{\alpha }$	Feature Time (s)	Optimize Time (s)
Adaptive-RCMDSE	0.6706	−2.3249	32.87	3294.67
Adaptive-TSMDSE	0.7023	−5.3373	57.51	5764.56
Adaptive-CMDSE	0.7457	−4.2419	31.42	3129.42
Adaptive-MDSE	0.7670	−1.4139	4.81	469.98
Adaptive-HDSE	0.7568	−3.0657	1.59	157.83

**Table 4:** Evaluation metrics for fault classification using different entropy features in Case II.

Feature Extraction Method	Optimal Accuracy (%)	Worst Accuracy (%)	Standard Deviation	Median Accuracy (%)	Mean Accuracy (%)
Adaptive-RCMDSE	100	99.00	0.0048	100	99.70
Adaptive-TSMDSE	99.00	95.00	0.0114	97.00	97.20
Adaptive-CMDSE	100	92.00	0.0251	97.50	96.50
Adaptive-MDSE	95.00	84.00	0.0374	91.50	90.30
Adaptive-HDSE	97.00	91.00	0.0207	95.00	94.60
MDE	97.00	91.00	0.0226	95.50	94.70
MDivE	97.00	91.00	0.0222	94.50	94.40
MBSIE	83.33	74.00	0.0295	80.00	79.50

**Table 5:** Evaluation metrics for Adaptive-RCMDSE and Adaptive-SVM optimized using different optimization algorithms in Case II.

Optimization Algorithm	Optimal Parameter Combination and Computational Cost				Fault Classification Recognition Rate (%)				
	Adaptive-RCMDSE		Adaptive-SVM		Optimal Accuracy	Worst Accuracy	Standard Deviation	Median Accuracy	Mean Accuracy
	$\alpha_{Best}$	Optimize Time (s)	$C_{Best}$,$\gamma_{Best}$	Optimize Time (s)					
LSC-SAO	0.6706	3294.67	91.0011, 19.0525	29.40	100	99.00	0.0048	100	99.70
SAO	0.6708	3429.34	77.6795, 6.5024	29.70	100	96.00	0.0148	99.50	98.80
MPA	0.6702	6895.66	47.1788, 9.1891	60.99	100	96.00	0.0129	99.00	98.90
WOA	0.6681	3441.18	100, 7.5710	29.98	100	97.00	0.0085	98.50	98.50
GWO	0.6722	3516.93	38.2928, 9.8628	29.42	100	94.00	0.0208	98.50	98.10

**Table 6:** Evaluation metrics for Adaptive-RCMDSE and Adaptive-SVM using different time series lengths in Case II.

Time Series Length	Optimal Parameter Combination and Key Performance Indicators						Fault Classification Recognition Rate (%)				
	LSC-SAO-RCMDSE			LSC-SAO-SVM			Optimal Accuracy	Worst Accuracy	Standard Deviation	Median Accuracy	Mean Accuracy
	$\alpha_{Best}$	Optimize Time (s)	Best Fitness	$C_{Best}$,$\gamma_{Best}$	Optimize Time (s)	Best Fitness					
2048	0.6706	3294.67	−2.3249	91.0011, 19.0525	29.40	0.0025	100	99.00	0.0048	100	99.70
4096	0.6703	5458.57	−4.8243	19.1746, 21.2171	30.02	0.0025	100	98.00	0.0067	100	99.70
8192	0.6867	9868.98	−10.0184	1.0551, 7.1003	29.55	0	100	100	0	100	100

**Table 7:** Description of seven bearing operating conditions in Case III.

Healthy Condition	Fault ID	Load ID	Label	Time Series Length	Number of Samples	Train Set Ratio	Test Set Ratio
Ball crack (B)	6205	00	1	2048	100	0.8	0.2
Inner race crack (I)	6205	00	2	2048	100	0.8	0.2
Outer race crack (O)	6205	00	3	2048	100	0.8	0.2
Normal (N)	6205	00	4	2048	100	0.8	0.2
Inner race and Ball cracks (IB)	6205	00	5	2048	100	0.8	0.2
Inner race and Outer cracks (IO)	6205	00	6	2048	100	0.8	0.2
Outer race and Ball cracks (OB)	6205	00	7	2048	100	0.8	0.2

**Table 8:** Key performance indicators of the fault feature extraction method for DSEs with adaptive control parameter in Case III.

Feature Extraction Method	$\alpha_{Best}$	Best $fitness_{\alpha }$	Feature Time (s)	Optimize Time (s)
Adaptive-RCMDSE	0.7665	−8.5039	43.53	4369.78
Adaptive-TSMDSE	0.8077	−32.4416	83.70	8303.30
Adaptive-CMDSE	0.7656	−16.8858	42.09	4217.94
Adaptive-MDSE	0.7845	−3.0804	6.70	663.60
Adaptive-HDSE	0.7813	−12.3886	2.23	221.48

**Table 9:** Evaluation metrics for fault classification using different entropy features in Case III.

Feature Extraction Method	Optimal Accuracy (%)	Worst Accuracy (%)	Standard Deviation	Median Accuracy (%)	Mean Accuracy (%)
Adaptive-RCMDSE	100	97.86	0.0082	99.64	99.29
Adaptive-TSMDSE	97.86	90.00	0.0222	94.29	94.21
Adaptive-CMDSE	100	97.14	0.0077	98.93	98.86
Adaptive-MDSE	92.86	84.29	0.0223	89.64	89.43
Adaptive-HDSE	92.86	87.14	0.0152	90.00	90.07
MDE	100	95.00	0.0158	97.86	97.86
MDivE	100	97.86	0.0089	99.64	99.29
MBSIE	92.14	85.71	0.0231	90.71	89.50

**Table 10:** Evaluation metrics for Adaptive-RCMDSE and Adaptive-SVM optimized using different optimization algorithms in Case III.

Optimization Algorithm	Optimal Parameter Combination and Computational Cost				Fault Classification Recognition Rate (%)				
	Adaptive-RCMDSE		Adaptive-SVM		Optimal Accuracy	Worst Accuracy	Standard Deviation	Median Accuracy	Mean Accuracy
	$\alpha_{Best}$	Optimize Time (s)	$C_{Best}$, $\gamma_{Best}$	Optimize Time (s)					
LSC-SAO	0.7665	4369.79	79.9745, 9.6338	56.01	100	97.86	0.0082	99.64	99.29
SAO	0.7663	4688.04	55.0683, 9.8124	57.38	100	97.86	0.0082	99.29	99.29
MPA	0.7890	9351.39	28.7464, 8.5440	114.16	100	96.43	0.0111	98.93	98.71
WOA	0.7664	4680.54	28.7464, 8.5440	56.71	100	97.14	0.0097	99.29	98.93
GWO	0.7640	4687.67	21.5005, 8.3638	56.12	100	96.43	0.0131	98.57	98.36

**Table 11:** Evaluation metrics for Adaptive-RCMDSE and Adaptive-SVM using different time series lengths in Case III.

Time Series Length	Optimal Parameter Combination and Key Performance Indicators						Fault Classification Recognition Rate (%)				
	LSC-SAO-RCMDSE			LSC-SAO-SVM			Optimal Accuracy	Worst Accuracy	Standard Deviation	Median Accuracy	Mean Accuracy
	$\alpha_{Best}$	Optimize Time (s)	Best Fitness	$C_{Best}$, $\gamma_{Best}$	Optimize Time (s)	Best Fitness					
2048	0.7665	4369.79	−8.5039	79.9745, 9.6338	56.01	0.0054	100	97.86	0.0082	99.64	99.29
4096	0.7594	7655.32	−16.8937	56.9537, 7.0029	62.74	0	100	100	0	100	100
8192	0.7530	13,782.22	−30.9154	89.9854, 98.0859	57.67	0	100	100	0	100	100

**Table 12:** Description of the nine health states of axle box bearings in Case IV.

Fault Type	Code	Fault Severity Level	Label	Time Series Length	Number of Samples	Train Set Ratio	Test Set Ratio
Cage fault	CC	Radial notch	1	2048	100	0.8	0.2
Inner face fault	IR	Level 1	2	2048	100	0.8	0.2
Normal	Nor	/	3	2048	100	0.8	0.2
Outer face fault	OR1	Level 1	4	2048	100	0.8	0.2
	OR2	Level 2	5	2048	100	0.8	0.2
	OR3	Level 3	6	2048	100	0.8	0.2
Rolling element fault	RE1	Level 1	7	2048	100	0.8	0.2
	RE2	Level 2	8	2048	100	0.8	0.2
	RE3	Level 3	9	2048	100	0.8	0.2

**Table 13:** Key performance indicators of the fault feature extraction method for DSEs with adaptive control parameter in Case IV.

Feature Extraction Method	$\alpha_{Best}$	Best $fitness_{\alpha }$	Feature Time (s)	Optimize Time (s)
Adaptive-RCMDSE	0.7482	−4.0984	59.69	5923.64
Adaptive-TSMDSE	0.7767	−4.5174	108.61	10,995.98
Adaptive-CMDSE	0.7407	−4.8745	56.41	5767.72
Adaptive-MDSE	0.8135	−1.4469	8.77	882.92
Adaptive-HDSE	0.8402	−2.3280	3.09	298.83

**Table 14:** Evaluation metrics for fault classification using different entropy features in Case IV.

Feature Extraction Method	Optimal Accuracy (%)	Worst Accuracy (%)	Standard Deviation	Median Accuracy (%)	Mean Accuracy (%)
Adaptive-RCMDSE	100	98.33	0.0053	99.44	99.28
Adaptive-TSMDSE	99.44	97.22	0.0079	98.89	98.67
Adaptive-CMDSE	100	97.78	0.0074	99.44	99.28
Adaptive-MDSE	95.56	95.56	0.0199	93.61	93.28
Adaptive-HDSE	95.00	90.56	0.0137	93.06	93.06
MDE	94.44	91.11	0.0115	92.50	92.83
MDivE	95.56	92.78	0.0080	94.17	94.17
MBSIE	46.11	34.44	0.0415	40.83	40.28

**Table 15:** Evaluation metrics for Adaptive-RCMDSE and Adaptive-SVM optimized using different optimization algorithms in Case IV.

Optimization Algorithm	Optimal Parameter Combination and Computational Cost				Fault Classification Recognition Rate (%)				
	Adaptive-RCMDSE		Adaptive-SVM		Optimal Accuracy	Worst Accuracy	Standard Deviation	Median Accuracy	Mean Accuracy
	$\alpha_{Best}$	Optimize Time (s)	$C_{Best}$, $\gamma_{Best}$	Optimize Time (s)					
LSC-SAO	0.7482	5923.64	48.0028, 4.9796	105.03	100	98.33	0.0053	99.44	99.28
SAO	0.7546	6028.03	38.2191, 10.2024	105.84	99.44	98.33	0.0045	98.89	98.89
MPA	0.7541	11,987.00	56.0973, 15.9306	202.65	99.44	97.22	0.0073	99.44	99.00
WOA	0.7552	5985.03	73.8735, 9.9019	100.29	100	97.78	0.0057	98.89	99.00
GWO	0.7475	5955.46	21.0122, 10.1392	105.03	100	96.67	0.0127	99.17	98.83

**Table 16:** Evaluation metrics for Adaptive-RCMDSE and Adaptive-SVM using different time series lengths in Case IV.

Time Series Length	Optimal Parameter Combination and Key Performance Indicators						Fault Classification Recognition Rate (%)				
	LSC-SAO-RCMDSE			LSC-SAO-SVM			Optimal Accuracy	Worst Accuracy	Standard Deviation	Median Accuracy	Mean Accuracy
	$\alpha_{Best}$	Optimize Time (s)	Best Fitness	$C_{Best}$, $\gamma_{Best}$	Optimize Time (s)	Best Fitness					
2048	0.7482	5923.64	−4.0984	48.0028, 4.9796	105.03	0.0069	100	98.33	0.0053	99.44	99.28
4096	0.8152	10,330.88	−8.0729	41.8121, 5.5134	100.56	0	100	98.89	0.0037	100	99.83
8192	0.8133	17,958.79	−15.1647	16.7274, 15.1651	99.69	0	100	100	0	100	100

## Data Availability

The data presented in this study are available upon request from the corresponding author upon request.
